# Genotypic and phenotypic consequences of domestication in dogs

**DOI:** 10.1093/g3journal/jkag195

**Published:** 2026-07-17

**Authors:** Sweetalana, Shirin Nataneli, Shengmiao Huang, Jazlyn A Mooney, Zachary A Szpiech

**Affiliations:** Department of Biology, Pennsylvania State University, University Park, PA 16802, United States; Department of Quantitative and Computational Biology, University of Southern California, Los Angeles, CA 90089, United States; Department of Quantitative and Computational Biology, University of Southern California, Los Angeles, CA 90089, United States; Department of Quantitative and Computational Biology, University of Southern California, Los Angeles, CA 90089, United States; Department of Biology, Pennsylvania State University, University Park, PA 16802, United States

**Keywords:** Runs of homozygosity, Dogs, Inbreeding, GWAS, Complex traits, PEQG2026

## Abstract

Runs of homozygosity (ROH) are genomic regions that arise when identical haplotypes are inherited from a shared ancestor. In this study, we explored ROH across 556 whole-genome sequences from domesticated and non-domesticated dogs. Then, we leveraged ROH from 466 breed dogs, representing 13 breed groups and 13 phenotypic traits, to investigate associations between genetic diversity and non-disease phenotypes. We identified significant associations between the ROH-based inbreeding coefficient (F_ROH_) and multiple phenotypes. These include three quantitative traits (height, weight, lifespan) and ten morphological and coat-related traits. After correcting for population structure, we identified more than 45 genes associated with quantitative traits that exceeded suggestive or genome-wide significance (GWS) thresholds. We also observed distinct patterns of inbreeding across dog populations, including elevated levels of long ROH in modern breed dogs relative to more ancient breeds, consistent with intensive breeding practices during Victorian-era breed formation. Together, our results demonstrate how domestication, demographic bottlenecks, and selective breeding have shaped patterns of homozygosity and contributed to the genetic architecture of complex traits in dogs, highlighting an important role for non-additive genetic variation and polygenicity.

## Teaser

This study is relevant to researchers interested in genetics, domestication, and complex traits. The authors investigated how inbreeding (measured by runs of homozygosity) and artificial selection have shaped the genome of dogs. They analyzed sequence data and quantify patterns of homozygosity across dog populations and tested for associations with non-disease traits in domestic (breed) dogs. Levels of homozygosity varied across domesticated and non-domesticated dogs and were associated with variation in height, weight, lifespan, musculature, and coat characteristics. These findings demonstrate how domestication and selective breeding have influenced genetic diversity and contributed to the genetic architecture of complex traits in dogs.

## Introduction

For over a century, scholars and dog enthusiasts alike have sought to unravel the complex evolutionary history of man's best friend (Darwin 1868). *Canis lupus familiaris* (dogs) have intrigued researchers across scientific fields due to their close association with humans (Nagasawa et al. 2015) and unique genetic features (Boyko 2011; Freedman et al. 2016; Perri et al. 2021). While the exact origins of domestication remain elusive, understanding how this process has shaped phenotypic variation and evolutionary history in dogs remains a central question.

In the nineteenth century, Darwin suggested that the broad range of features exhibited in modern breeds is consistent with descent from multiple canid species, including wolves and jackals. In his work on variation, he argued that a single ancestral origin from the gray wolf (*Canis lupus*) conflicted with the historical and archaeological evidence available at the time. Given that many *Canis* species can interbreed, Darwin's proposal held fast for many years (Darwin 1868). Advances in genomic technology have since provided new approaches to studying dog ancestry, offering insights that complement and, in some cases, challenge conclusions drawn from archaeological and historical evidence (Gray 1972). One commonly used approach is mitochondrial DNA (mtDNA) analysis, which allows researchers to trace maternal ancestry. Using samples from dogs, wolves, and several other wild canid species, these studies found that dogs are genetically more similar to gray wolves than to any other canid species (Wayne 1993; Vilà et al. 1997). More recent whole-genome sequencing (WGS) studies have further clarified that wolves are the closest relatives of dogs, making their evolutionary relationship a major focus of contemporary research (Freedman et al. 2016; Meadows et al. 2023).

While most agree that dogs were the first domesticated species, establishing their spatial and temporal roots remains a challenge (Larson and Bradley 2014). Early depictions of human–canine ties come from cave paintings discovered in Saudi Arabia (Guagnin et al. 2018). These cooperative hunting scenes are estimated to be 8,000–9,000 years old, but findings from burial sites suggest an even earlier origin of domestication (Morey 2006). Archaeological evidence dates to approximately 12,000 years ago to the Natufian in northern Israel, where three sets of dog remains were found buried alongside a human (Davis and Valla 1978; Tchernov and Valla 1997). Additionally, a doglike mandible recovered from a burial site in Bonn-Oberkassel, Germany, has been dated to approximately 14,000 years ago (Nobis 1979; Benecke 1987).

Genetic research provides an additional line of evidence suggesting an even earlier wolf–dog divergence. Studies based on mtDNA and WGS data have proposed two primary models for the origins of domestication. The first posits a single domestication event from gray wolves in East Asia between 16,000 and 100,000 years ago. The hypothesis is supported by limited haplotypic diversity in dogs, consistent with a genetic bottleneck in which domesticated dogs originated from a small, closely related population of gray wolves (Vilà et al. 1997). High levels of genetic diversity in regions south of the Yangtze River further suggest an earlier divergence relative to other parts of Europe and Asia (Savolainen et al. 2002; Pang et al. 2009; Wang et al. 2016).

The second genomic narrative centers on the hypothesis that haplogroups entered the dog lineage at different times and locations. Using nuclear genomic single nucleotide polymorphism (SNP) analysis, one study found evidence for allele sharing between Asian dog breeds and Asian wolves, as well as between European dog breeds and European wolves (vonHoldt et al. 2010). These findings suggest multiple founder events derived from distinct wolf populations. In addition, WGS studies have documented substantial genetic differences between Eastern and Western Eurasian dog populations, further supporting multiple domestication events (Fan et al. 2016; Bergström et al. 2022). Despite these findings, the origins of dog domestication remain a hot topic of debate, highlighting the need for higher-resolution genomic data and improved temporal and geographic sampling to better resolve the timing, location, and number of domestication events.

While the early evolution of dogs from wolves marked a significant shift between species, the more recent history of breed formation has shaped evolution within the species itself. During the Victorian era (nineteenth century), selective breeding for aesthetically desirable traits led to the emergence of hundreds of new breeds. Breed establishment typically involved a limited number of founding individuals, resulting in high levels of inbreeding and considerable loss of genetic diversity (Boyko 2011; Cecchi et al. 2013; Mastrangelo et al. 2018; Yordy et al. 2020; Bannasch et al. 2021). Such founder effects restrict the gene pool and produce long stretches of shared DNA between individuals. Segments inherited from a single common ancestor are referred to as identical-by-descent (IBD). Within an individual, inheriting the same IBD segment from both parents creates runs of homozygosity (ROH). Artificial selection for extreme phenotypes during breed formation resulted in large portions of the genome falling within ROH, simplifying complex trait architecture in breed dogs and facilitating the study of phenotypic associations between breeds (Boyko 2011; Plassais et al. 2019; Sams and Boyko 2019).

Studies of ROH in humans have revealed associations with numerous complex disease and non-disease phenotypes, offering valuable insight into the impact of genetic diversity on health and evolutionary processes (Bacolod et al. 2008; Keller et al. 2012; Pemberton 2012; Pemberton and Szpiech 2018; Clark et al. 2019; Mooney et al. 2021; Moreno-Grau et al. 2021). Similar work in dogs has identified associations between ROH and several disease phenotypes (Boyko 2011; Sams and Boyko 2019; Mooney et al. 2021). Additionally, previous studies have examined the influence of genetic variants on quantitative traits such as leg length, wrinkled skin, coat color, hair length, and skeletal shape (Candille et al. 2007; Sutter et al. 2007; Cadieu et al. 2009; Parker et al. 2009; Boyko et al. 2010). Despite these advances, associations between ROH and complex non-disease phenotypes in dogs remain relatively unexplored (Hayward et al. 2016).

In this study, we investigate the relationship between the genomic consequences of domestication and complex (non-disease) trait architecture by leveraging ROH distributions to study phenotypic variation. We analyzed 556 canid whole-genome sequences to characterize the genomic distribution of ROH, and after accounting for breed structure, tested for associations between total ROH coverage (F_ROH_) across 466 breed dogs that spanned 13 breed groups and 13 phenotypes. We further performed an ROH-mapping genome-wide association study (GWAS) to identify genetic variants associated with height, weight, and lifespan. Beyond providing insight into dog domestication, this framework highlights how ROH-based approaches can be a powerful approach to study genotype–phenotype relationships in populations that experienced bottlenecks, inbreeding, or strong selection.

## Methods

### Clustering for clade-level dendrogram

Data from (Mooney et al. 2021) was filtered to an overlapping set of 84 groups of *N* = 2,822 village and breed dogs. Some, but not all, of these samples were shared with our dataset. Pairwise genetic relatedness was estimated using a kinship matrix generated with PC-Relate. Kinship values were normalized to a zero to one scale. Individuals were labeled with their known breed and clade assignment. Clade-level relatedness was computed as the mean normalized kinship across all pairwise comparisons between individuals from each pair of clades, resulting in a symmetric similarity matrix. This matrix was converted to a distance matrix. Hierarchical clustering was performed using the “ward.D2” method implemented in the *hclust* function in R. Dendrograms were generated from the resulting clustering and visualized using the ggdendro package (De Vries and Ripley 2025).

### Data filtering and categorization

We analyzed whole-genome sequence data from 722 canids, including wild species, dingoes, and domestic dogs (Plassais et al. 2019). These data are publicly available under NCBI accession number PRJNA448733. Variant filtering was performed using BCFtools (Danecek et al. 2021). We retained only biallelic SNPs with genotype quality (GQ) score ≥ 20 and excluded sites with > 10% missing genotypes. Variants located on sex chromosomes were removed.

After filtering, the dataset comprised 4,053,761 biallelic loci across the 38 autosomes. To focus on breed-level analyses, we omitted wild canids, mixed-breed individuals, and samples lacking breed annotations. The remaining samples were categorized by common name or breed (Yordy et al. 2020). These classifications were informed by neighbor-joining trees of domestic dogs (vonHoldt et al. 2010) and American Kennel Club (AKC) groupings (Dog Breeds—Types Of Dogs 2024 Oct 14). This resulted in 13 breed groups: ancient spitz (31 individuals and 11 breeds), herding (76 individuals and 15 breeds), mastiff-like (65 individuals and 15 breeds), retrievers (50 individuals and 6 breeds), scent hound (23 individuals and 10 breeds), small terrier (102 individuals and 8 breeds), terriers (16 individuals and 7 breeds), spaniels (22 individuals and 8 breeds), toy dogs (18 individuals and 7 breeds), working dogs (44 individuals and 10 breeds), sight hound (21 individuals and 9 breeds), village dogs (75 individuals across 13 regions), and Chinese indigenous dogs (15 individuals). Overall, the final dataset included 558 individuals representing 106 different breeds (Supplementary Table 1).

### Calling runs of homozygosity (ROH)

ROH were identified using GARLIC v1.6.0a, which uses a likelihood-based inference framework (Szpiech et al. 2017). This approach infers autozygosity using a logarithm of odds (LOD) score calculated within a sliding window across the genome (Pemberton 2012). GARLIC requires genotype and sample information in TPED and TFAM formats, which were generated using PLINK 2.0 (Chang et al. 2015). A TGLS file was also generated to obtain per-genotype likelihoods. Genotype likelihoods were derived from GQ scores to account for uncertainty in phred-scaled probabilities. A window size of 100 SNPs was selected based on SNP density (Supplementary Fig. 28), with the window advancing in increments of 10 SNPs at each step. GARLIC's built-in ROH length classification was used to define six classes: <1 Mb, 1–2 Mb, 2–3 Mb, 3–4 Mb, 4–5 Mb, and >5 Mb. To reduce noise from short, common segments, only ROH segments longer than 0.5 Mb were retained. To account for sample size differences in allele frequency estimation, we set the number of resamples to 40. All other parameters were left at default settings. The following flags were used: “–auto-winsize –auto-overlap-frac –winsize 100 –centromere –size-bounds 1000000 2000000 3000000 4000000 5000000 –tped –tfam –tgls –gl-type GQ –resample 40 –out.”

ROH calling was performed independently for breeds with at least 10 individuals, whereas those with smaller sample sizes were assigned to their respective breed groups prior to inference (Supplementary Table 2). Subsequently, ROH calls from all breeds, irrespective of sample size, were aggregated at the breed group level for downstream analysis. For each individual, we examined the relationship between coverage and F_ROH_ (Supplementary Fig. 29) and the relationship between the total number of ROH segments (n_ROH_) and total ROH length (s_ROH_) (Supplementary Fig. 30). For each individual and breed group, we calculated the arithmetic mean and range of total n_ROH_ and total s_ROH_, stratified by ROH length class.

Two individuals exhibited extreme values: PER00747 showed the longest total s_ROH_ (2141.22 Mb), and PER00393 the highest total nROH (1039), both from the small terrier Yorkshire breed. PER00747 was sequenced at a low depth (∼2x), which likely inflated s_ROH_ estimates. To mitigate the effects of low depth and extreme values, both PER00747 and PER00393 were excluded from downstream analyses, resulting in a final sample size of *N* = 556.

### Inbreeding coefficient

For each individual and breed group, we computed an ROH-based inbreeding coefficient, F_ROH_. F_ROH_ is the fraction of the autosomal genome contained within ROH regions (McQuillan 2008):


$$F_{ROH}=\;\frac{s_{ROH}}{L_{Auto}}$$

Here, s_ROH_ denotes the total length of ROH segments in an individual genome, and L_Auto_ represents the total length of the autosomal genome in base pairs. We used an autosomal genome length of 2,203,764,842 base pairs, corresponding to the CanFam3.1 reference genome (NCBI RefSeq assembly: GCF_000002285.3), from which variants were called.

### ROH sharing matrix

We constructed an ROH sharing matrix where each entry represents the total length of overlapping ROH segments between a pair of individuals. This approach was used to quantify patterns of shared autozygosity across dogs. To generate these values, ROH segments for each sample were converted into BED format. Pairwise intersections were then identified using BEDTools (Quinlan and Hall 2010) with the “intersect -wao” option, which reports all shared regions while retaining nonoverlapping intervals. The length of shared ROH segments was obtained by summing overlap lengths across all intersecting intervals for each pair of individuals.

### Phenotype data

We updated the phenotypic data (Plassais et al. 2019) for 13 traits: bulky, drop ears, furnish, hairless, height, large ears, length of fur, lifespan, long legs, muscled, weight, white chest, and white head. For three continuous traits (height, weight, and lifespan), we used breed–average values obtained from the AKC (Dog Breeds—Types Of Dogs 2024 Oct 14) (Supplementary Table 3). These values were used because they have been shown to be reliable for association analyses (Jones et al. 2008; Boyko et al. 2010; Rimbault et al. 2013; Hayward et al. 2016; Plassais et al. 2017, 2019). When sex-specific information was available, it was incorporated; otherwise, the same values were assigned to both males and females.

Continuous traits were normalized using min–max scaling, and dogs were categorized into small (less than) and large (greater than) groups based on the mean weight (21.66 kg) across all individuals (Supplementary Tables 4 to 6). This threshold provides a straightforward and reproducible partition of dogs into size classes that differ in life history traits such as growth, metabolism, and lifespan. Body size is correlated with breed structure, and our grouping approach, which is based on the mean weight across breeds, is a simplification of continuous body size variation.

For the remaining quantitative traits, breed-level averages were calculated based on the available phenotypic data (Supplementary Table 7). Lastly, binary encoding was applied, assigning a value of 1 to individuals expressing the trait and 0 otherwise.

### Association tests

We restricted the dataset to (*N* = 466) breed dogs, excluding Chinese indigenous dogs and village dogs due to missing phenotype data. Associations between F_ROH_ and 13 phenotypes were tested using GMMAT, an R package for generalized linear mixed model (GLMM) analyses (Chen et al. 2016). Models were fit using the glmmkin function, incorporating a kinship matrix derived from pairwise ROH sharing between individuals. This approach controls for population structure and relatedness in a manner analogous to genetic relationship matrix (GRM)-based approaches used in standard GWAS (Mooney et al. 2021).

For continuous traits (height, weight, and lifespan), phenotypes were modeled as the response variable under a Gaussian distribution with an identity link function, appropriate for continuous outcomes. Breed–average phenotypic values were used for all 466 individuals, as this approach provides a reliable approximation to individual-level associations, as demonstrated through simulations and theoretical justification (Supplementary Fig. 31 and Supplementary Text). We fit a series of linear regression models testing associations between F_ROH_ and height, weight, and lifespan in the full (all) breed dog dataset (*n* = 466) and in small- and large-bodied subsets. Models included univariable F_ROH_ effects, covariate models (weight or lifespan), and interaction models including F_ROH_ × weight terms to assess potential body-size-dependent effects of inbreeding (Supplementary Table 8). We included these covariates and interaction terms because previous studies demonstrated associations between body size, inbreeding, and lifespan (Fareed and Afzal 2014; Kim et al. 2018; Yordy et al. 2020).

For the remaining 10 binary traits, GLMMs were fit using the binomial distribution with a logit link function, appropriate for modeling binary outcomes (Supplementary Table 9). Samples lacking phenotype information were excluded from the corresponding analyses.

### ROH-mapping GWAS

To investigate the genetic basis of ROH-associated variation in phenotypic traits, we conducted GWAS. We generated a dataset of SNPs located within ROH regions using BEDTools intersect and subtract (Quinlan and Hall 2010), retaining only variants that overlapped ROH segments.

Association analyses were performed using GMMAT under the same GLMM framework described for the association tests, incorporating the kinship matrix derived from pairwise ROH sharing between individuals to account for relatedness, consistent with previous studies of inbreeding and trait variation in dogs (Mooney et al. 2021). For quantitative traits (height, weight, and lifespan), ROH-associated SNPs were modeled as predictors under a recessive genetic model, assuming a Gaussian distribution with an identity link function (Chen et al. 2016). Weight and the interaction between weight and F_ROH_ were included as covariates across the three data subsets (all individuals, small individuals, and large individuals) (Supplementary Table 10). To account for autocorrelation among SNPs due to linkage disequilibrium (LD), we estimate the effective sample size, using the *effectiveSize* function in the coda R package (Plummer et al. 2006), using genome-wide *P*-values as input. This approach was shown to be effective for accounting for correlation structure among SNPs and resulting test statistic inflation (Shriner et al. 2011).

Genome-wide significance and suggestive-wide significance (SWS) thresholds were determined using Bonferroni correction based on effective sample size (Li et al. 2012; Yang et al. 2014; Yuan et al. 2020):

Genome-wide significance threshold $=\;-log_{10}\left(\frac{0.05}{Effective\;Sample\;Size}\right)$

Suggestive-wide significance threshold $=\;-log_{10}\left(\frac{0.1}{Effective\;Sample\;Size}\right)$

To assess residual population stratification, we calculated the genomic inflation factor, λ (Van Den Berg et al. 2019; Sofer et al. 2021), and adjusted test statistics accordingly (Supplementary Table 10). Genome-wide association study summary statistics were visualized using the qqman R package to generate quantile–quantile and Manhattan plots (Turner 2018), with results shown in Supplementary Figs. 17 to 25.

## Results

**Fig. 1.**
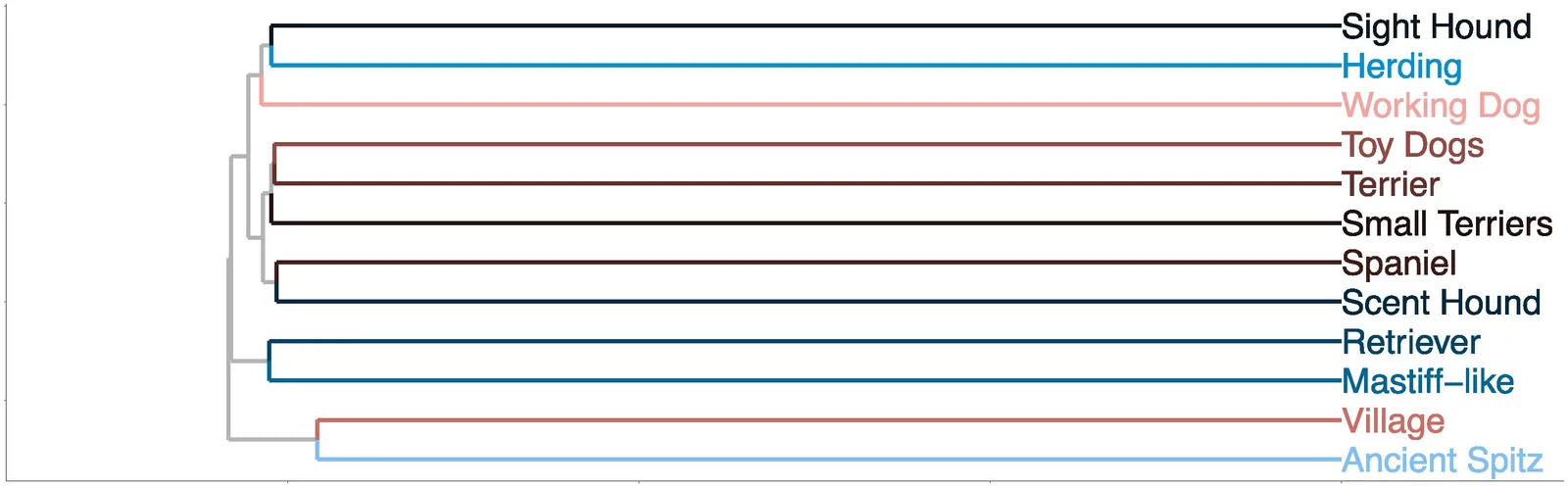
Clustering dendrogram of clade-level genetic relationships. Hierarchical clustering was conducted on the kinship matrix of a larger set of dogs that were shared with (Mooney et al. 2021). Each branch is a different color that corresponds to distinct clade identity. Internal branches reflect shared ancestry across multiple clades. Clades that cluster more closely share higher genetic similarity, while clades that are further apart have greater genetic divergence.

Breed dogs were categorized into clades (Fig. 1) for most analyses, and for the association tests, dogs were stratified by body size (see *Methods*). Clade-level relationships are consistent with previous studies (vonHoldt et al. 2010; Meadows et al. 2023), with village dogs and ancient spitz breeds representing the most divergent groups relative to Victorian-era breeds. We characterized these relationships by constructing a dendrogram based on kinship estimates from (Mooney et al. 2021). This dataset was selected due to substantial overlap with our samples (excluding Chinese indigenous dogs) and its larger sample sizes. In total, we analyzed 556 individuals, including both breed and non-breed dogs (Chinese indigenous dogs and village dogs). Association analyses were performed on a subset of 466 breed dogs with available phenotypic data.

### ROH distribution

**Fig. 2.**
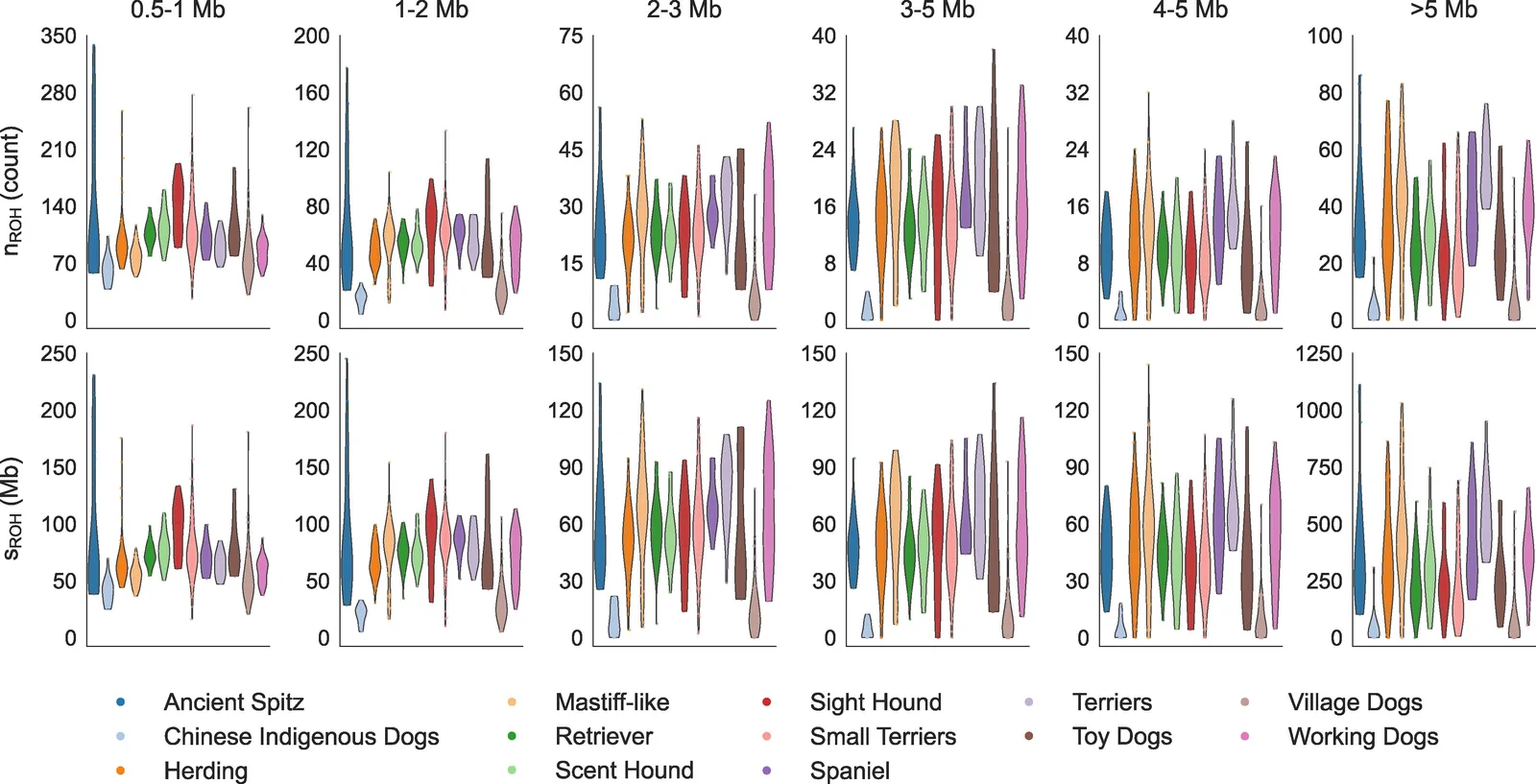
Distribution of ROH across dog clades and length classes. Violin plots show the distribution of the number of ROH (n_ROH_; top row) and total ROH length (s_ROH_; bottom row, Mb) across 12 dog clades for six ROH length classes (0.5–1 Mb, 1–2 Mb, 2–3 Mb, 3–4 Mb, 4–5 Mb, and >5 Mb). Each violin represents the distribution across individuals within a clade, with colors corresponding to breed groups. Short ROH are common across all clades, whereas long ROH (>5 Mb) show greater variation among clades and are observed more frequently in domestic breed groups relative to Chinese indigenous and village dogs.

ROH were called across the full dataset of 556 individuals, including both breed and non-breed dogs. ROH were categorized into six length classes: Class A (0.5–1 Mb), Class B (1–2 Mb), Class C (2–3 Mb), Class D (3–4 Mb), Class E (4–5 Mb), and Class F (>5 Mb). Across all classes, the mean number of ROH (n_ROH_) was 37.06 and the mean total length (s_ROH_) was 94.17 Mb. This pattern was largely driven by breed dogs (Fig. 2), which vary in both genetic diversity and ROH profiles across clades. Shorter tracts (0.5–2 Mb) were abundant across all groups and showed less variance than longer tracts (>5 Mb), which were more variable in both abundance and total genomic coverage.

Domestic breed groups generally exhibited a greater abundance and total length of long ROH (>5 Mb), whereas Chinese indigenous dogs and village dogs tended to have fewer long ROH and less of their genomes covered by ROH. Among breed dogs, ancient spitz individuals exhibited the highest total n_ROH_ (641) and s_ROH_ (1643.98 Mb) (Supplementary Table 11). In contrast, terriers showed the lowest mean n_ROH_ and s_ROH_ among the 11 remaining breed groups. Lastly, mean n_ROH_ and s_ROH_ were computed for each length class and are summarized in Supplementary Tables 12 and 13.

**Fig. 3.**
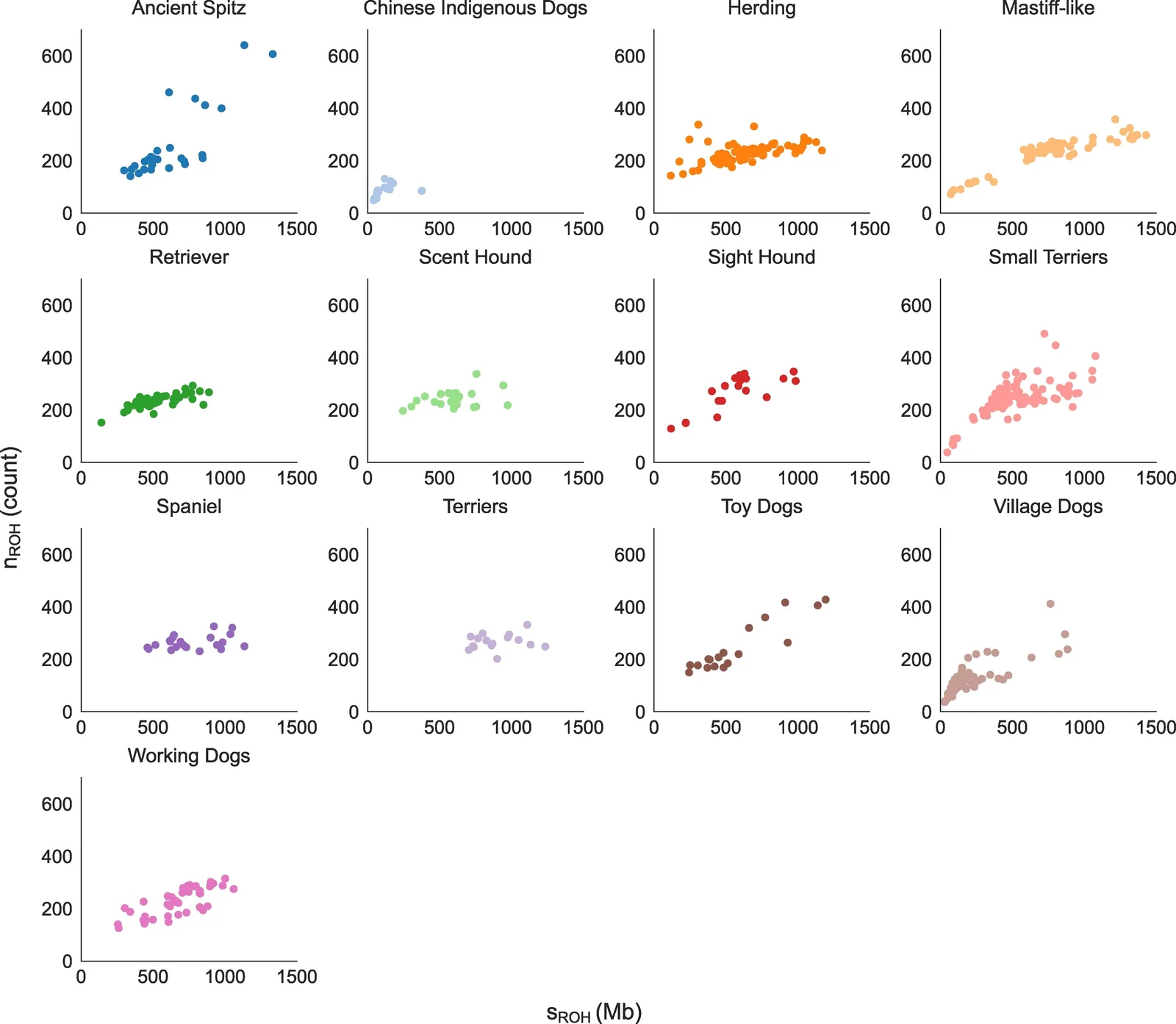
Relationship between ROH length and ROH count across dog breed groups. Each panel shows individual dogs within a breed group, plotting total ROH length (s_ROH_, Mb) against the number of ROH segments (n_ROH_). Points represent individual genomes. Axes are standardized across panels to facilitate comparison. Variation in clustering and spread reflects differences in ROH profiles among breeds, consistent with variation in inbreeding levels and demographic history.

We explored the relationship between the total number of ROH (n_ROH_) and the cumulative length of ROH (s_ROH_) (Fig. 3). Domesticated breed dogs exhibited higher mean values for both n_ROH_ and s_ROH_ compared to non-breed populations, including Chinese indigenous dogs and village dogs. However, patterns of n_ROH_ vs s_ROH_ varied across clades. The terrier clade was in the upper range for both n_ROH_ and s_ROH_ values, consistent with their relatively high mean F_ROH_ values reported in Table 1. Small terriers had a large amount of variance in n_ROH_ values across breeds relative to other clades (Fig. 3). Ancient Spitz, village dogs, herding, mastiff-like, and toy dogs also exhibited a broad variance when comparing n_ROH_ vs s_ROH_. In contrast, Chinese indigenous dogs, terriers, scent hounds, and retrievers formed relatively tight clusters when comparing n_ROH_ vs s_ROH_. Spaniels exhibited intermediate patterns between these extremes. Overall, differences in clustering and dispersion across clades highlighted variation of homozygosity within the breeds that form each clade, with some clades having more heterogeneous distributions of homozygosity within the n_ROH_ vs s_ROH_ space.

**Table 1.** F_ROH_ across all breed groups.

Breed group	Mean F_ROH_
Terriers	0.41 ± 0.07
Spaniels	0.35 ± 0.09
Mastiff-like	0.34 ± 0.15
Ancient spitz	0.33 ± 0.17
Working dog	0.31 ± 0.09
Herding	0.29 ± 0.11
Scent hound	0.27 ± 0.08
Toy dogs	0.26 ± 0.13
Sight hound	0.26 ± 0.10
Small terrier	0.24 ± 0.10
Retriever	0.24 ± 0.07
Village dogs	0.09 ± 0.08
Chinese indigenous dogs	0.05 ± 0.04

Values represent the mean (± standard deviation) of the F_ROH_ for each clade of dog.

We investigated whether ROH hotspots were shared across clades or between breed and non-breed populations. For each chromosome, we calculated ROH coverage per site and identified regions where more than 50% of individuals within a clade carried a ROH (Supplementary Figs. 1 to 16). ROH hotspots were present across all clades and chromosomes. Some hotspots occurred in regions with varying recombination rates. For example, on chromosome 22, shared ROH hotspots coincided with regions of lower recombination rates. In contrast, chromosome 12, which exhibits higher recombination rates, showed relatively few shared hotspots.

Shared ROH hotspots across clades were rare. Notable exceptions include chromosome 28, where a hotspot was shared among ancient spitz, mastiff-like, retrievers, and terriers (Supplementary Fig. 13), and chromosome 32, where overlap was observed among mastiff-like, scent hounds, terriers, and toy dogs (Supplementary Fig. 14). More commonly, hotspots were clade-specific, with only a small number of regions showing overlap among multiple clades. For example, chromosome 17 contained multiple clade-specific ROH peaks (Supplementary Fig. 9). Similar patterns were seen on chromosomes 31–38, with occasional sharing between mastiff-like breeds and terriers (Supplementary Figs. 14 to 16). Chinese indigenous dogs and village dogs showed fewer ROH hotspots than most breed groups (Supplementary Figs. 3 to 16).

We quantified consanguinity within clades using the ROH-based inbreeding coefficient, F_ROH_. We computed F_ROH_ for each individual and summarized values by clade (Table 1 & Supplementary Table 14). Mean F_ROH_ values were consistently higher in breed dogs compared to non-breed populations. Among breed groups, terriers exhibited the highest mean F_ROH_ (0.41 ± 0.07), while retrievers showed the lowest (0.24 ± 0.07). In contrast, substantially lower mean F_ROH_ values were observed in non-breed populations, including Chinese indigenous dogs (0.05 ± 0.04) and village dogs (0.09 ± 0.08).

### Association between F_ROH_ and phenotypic traits

**Fig. 4.**
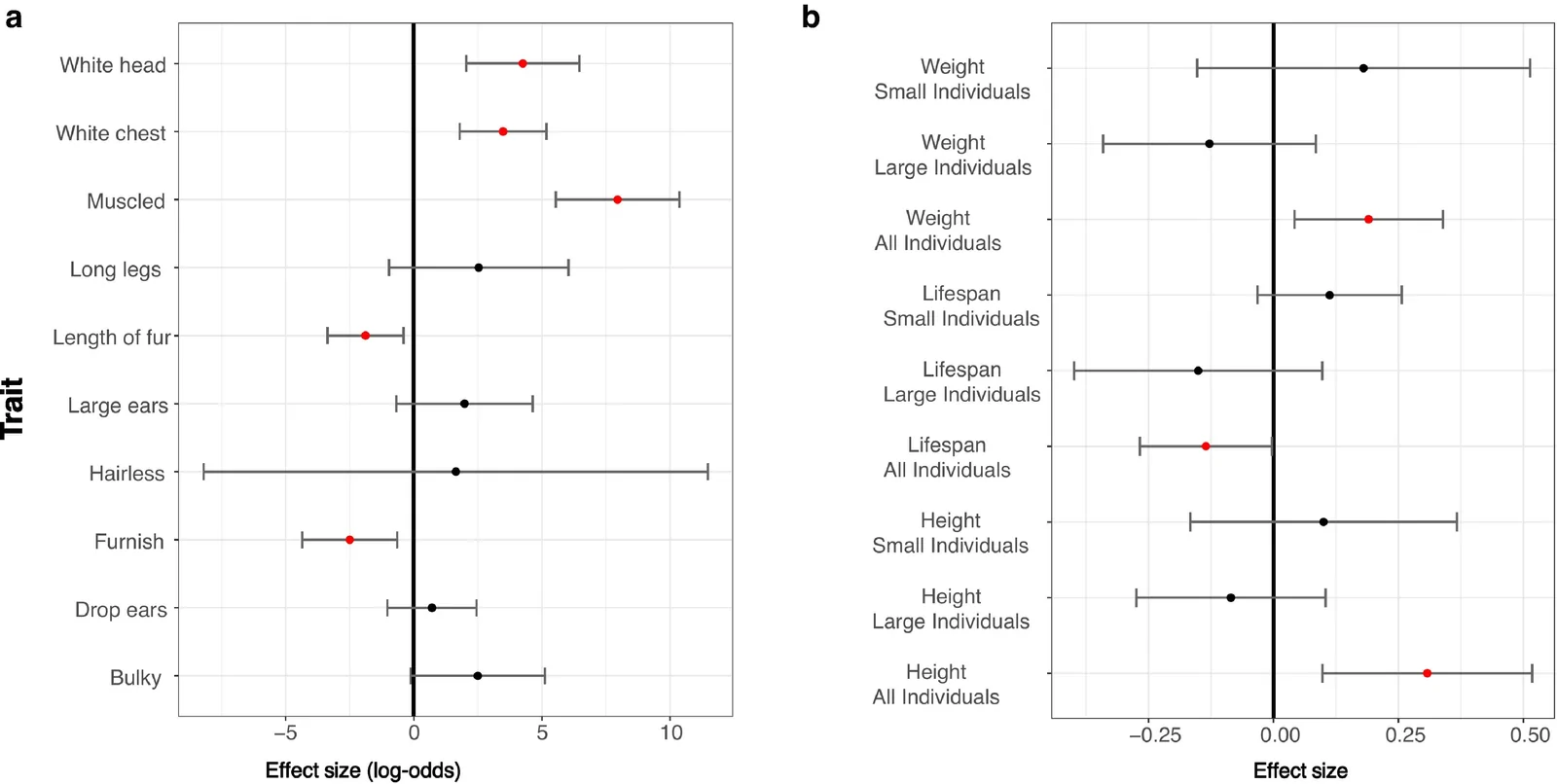
Relationship between effect size and (a) binary or (b) breed–average phenotypic traits. The *y*-axis shows the phenotypes and how individuals were groups. The *x*-axis shows log-odds effect sizes or normalized effect sizes (β) for the association between F_ROH_ and each trait. Points represent effect size estimates and horizontal bars indicate confidence intervals. Statistically significant associations (*P* < 0.05) are highlighted in red. (a) Positive effect sizes indicate an increase in the odds of the trait with increasing F_ROH_, whereas negative values indicate a decrease. (b) Positive effect sizes indicate an increase in trait value with increasing F_ROH_, whereas negative values indicate a decrease.

We then restricted the dataset to breed dogs and examined whether F_ROH_ could be used to detect non-additive effects in quantitative (non-disease) traits (Fig. 4). Following approaches previously applied to disease phenotypes in dogs (Mooney et al. 2021) and both quantitative and disease phenotypes in humans (Johnson et al. 2018; Clark et al. 2019; Barton et al. 2022; Lynch et al. 2023; Malawsky et al. 2023; Chundru et al. 2024; Fridman et al. 2025), we tested for non-additive effects by correlating F_ROH_ with 13 breed–average traits (Fig. 4). These included bulky build, drop ears, furnish, hairlessness, height, large ears, length of fur, lifespan, long legs, muscled build, weight, white chest, and white head. Furnish refers to the presence of facial hair (eg eyelashes, mustache, and beard) in dogs.

Five of the ten binary phenotypes we tested showed significant associations with F_ROH_: muscled (β=7.96 and $p=9.68\times \;10^{-11}$), white chest (β=3.49 and $p=5.36\times \;10^{-5}$), white head (β=4.26 and $p=1.55\times \;10^{-4}$), length of fur (β=−1.88 and $p=1.27\times \;10^{-2}$), and furnish (β=−2.50 and $p=8.24\times \;10^{-3}$) (Fig. 4a, Supplementary Table 9). Across breed groups, higher F_ROH_ was associated with increased prevalence of muscled, white chest, and white head phenotypes, whereas lower F_ROH_ was associated with increased prevalence of length of fur and furnish phenotypes.

All three quantitative traits showed significant associations with F_ROH_: breed–average height (β=0.31 and $p=4.05\times 10^{-3}$), breed–average weight (β=0.19 and $p=1.19\times \;10^{-2}$), and breed–average lifespan (β=−0.14 and $p\;=4.44\times \;10^{-2}$) (Fig. 4b). In breed dogs, a 1% increase in F_ROH_ was associated with an increase of 0.192 cm in breed–average height and 0.14 kg in breed–average weight and a decrease of 0.014 years in breed–average lifespan. Full statistical results, including interaction terms, normalized effect sizes (*β* or log-odds), *P*-values, and confidence intervals for quantitative traits, are provided in Supplementary Table 8.

### ROH-mapping GWAS

Finally, we sought to identify ROH-associated genomic regions influencing quantitative traits of interest (height, weight, and lifespan). We performed a GLMM-based GWAS (Figs.5 to 7 and Supplementary Figs. 17 to 25), using the presence or absence of ROH at each locus across individuals as the predictor. Significance thresholds are reported in Supplementary Table 10.

**Fig. 5.**
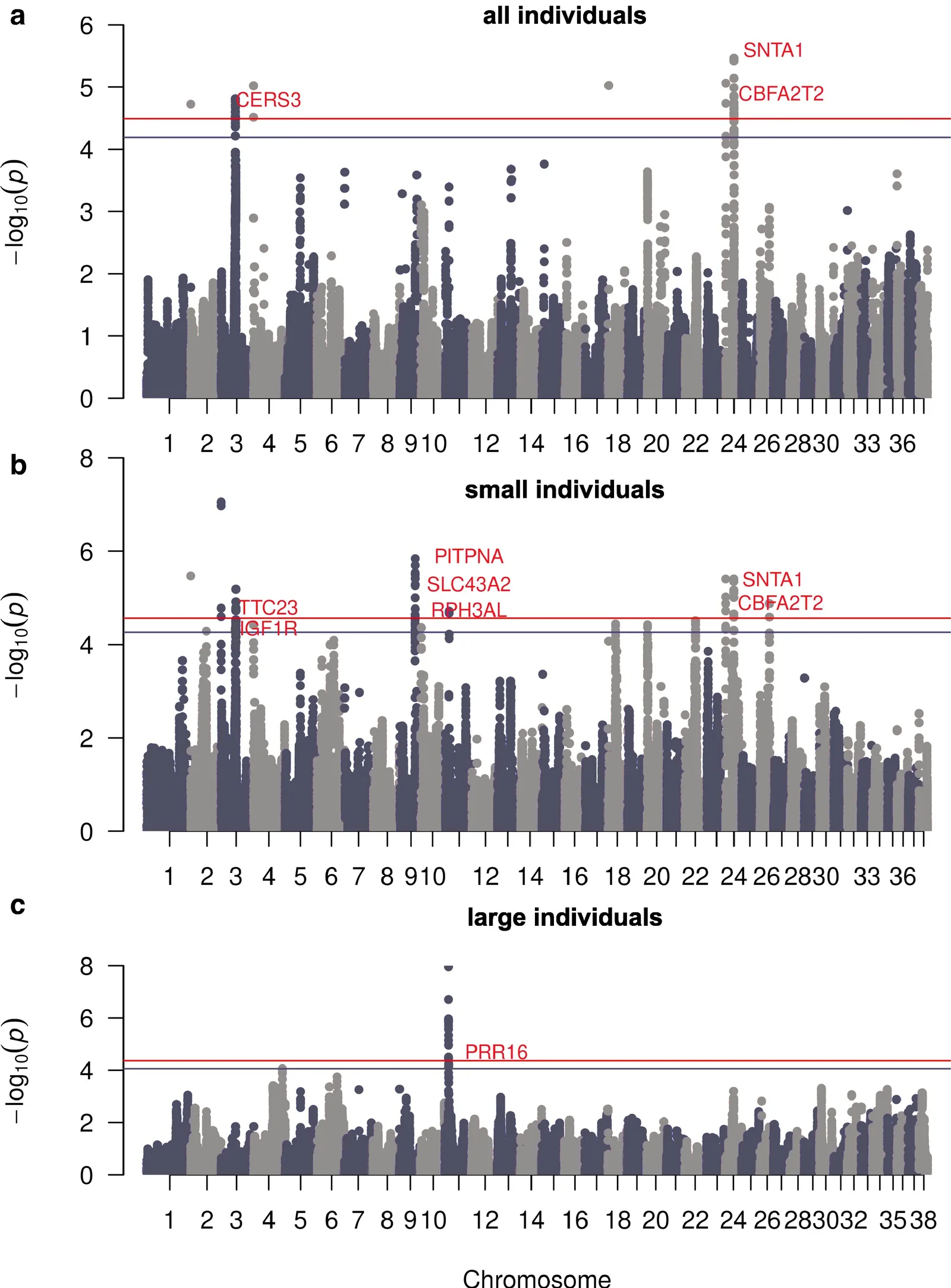
Manhattan plots for GLMM-based GWAS of ROH-associated loci for height. Panels show results for (a) all individuals, (b) small individuals, and (c) large individuals. The *x*-axis shows genomic position by chromosome, and the *y*-axis shows $-log_{10}$(*P*-values). Each point represents a single nucleotide polymorphism (SNP). The red horizontal line indicates the genome-wide significance (GWS) threshold, and the blue horizontal line indicates the suggestive-wide significance (SWS) threshold. SNPs exceeding the GWS threshold are labeled, apart from IGF1R. Gene labels are not drawn to scale.

**Fig. 6.**
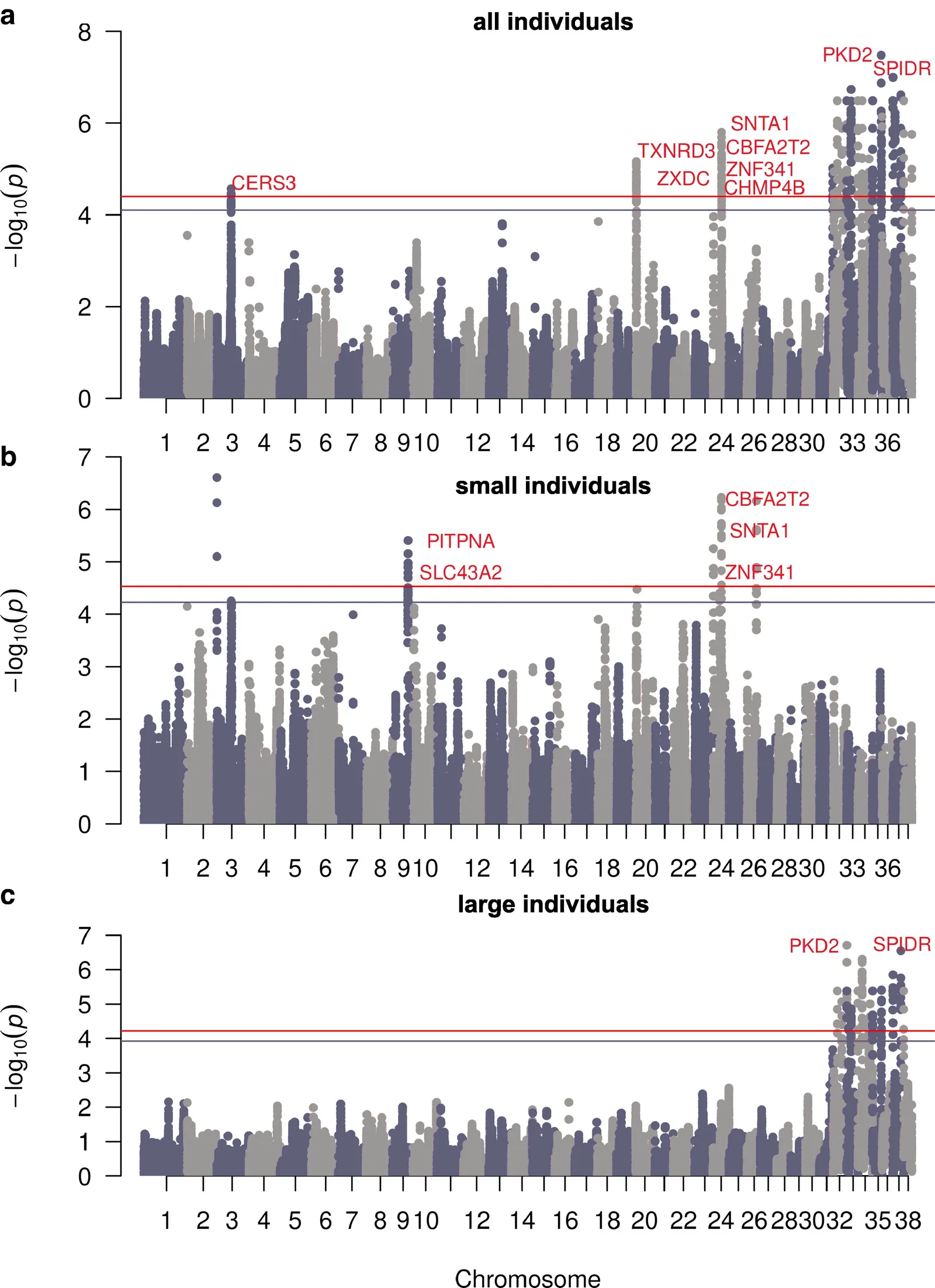
Manhattan plots for GLMM-based GWAS of ROH-associated loci for weight. Panels show results for (a) all individuals, (b) small individuals, and (c) large individuals. The *x*-axis shows genomic position by chromosome, and the *y*-axis shows $-log_{10}$(*P*-values). Each point represents a single nucleotide polymorphism (SNP). The red horizontal line indicates the genome-wide significance (GWS) threshold, and the blue horizontal line indicates the suggestive-wide significance (SWS) threshold. SNPs exceeding the GWS threshold are labeled. Gene labels are not drawn to scale.

**Fig. 7.**
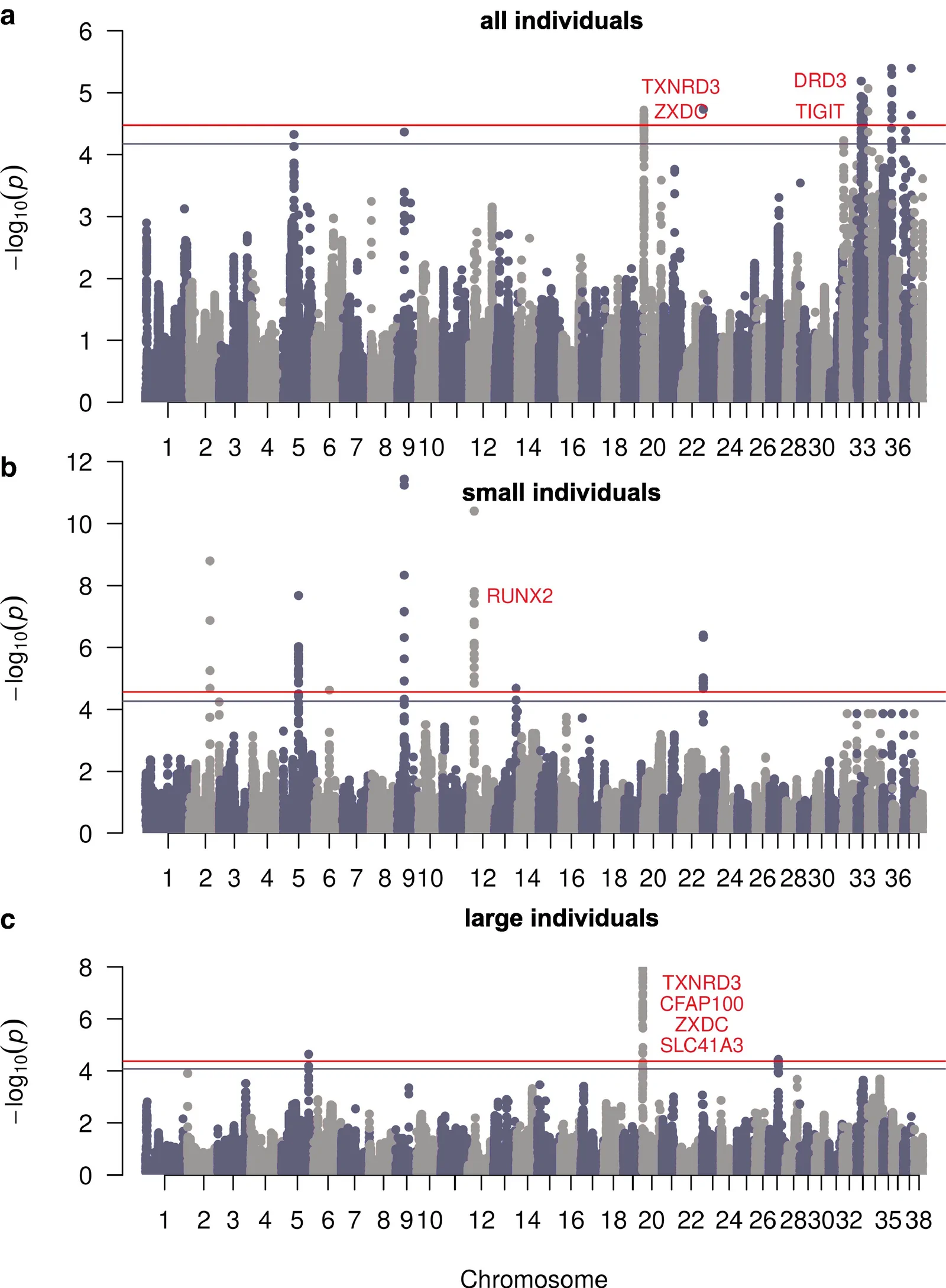
Manhattan plots for GLMM-based GWAS of ROH-associated loci for lifespan. Panels show results for (a) all individuals, (b) small individuals, and (c) large individuals. The *x*-axis shows genomic position by chromosome, and the *y*-axis shows $-log_{10}$(*P*-values). Each point represents a single nucleotide polymorphism (SNP). The red horizontal line indicates the genome-wide significance (GWS) threshold, and the blue horizontal line indicates the suggestive-wide significance (SWS) threshold. SNPs exceeding the GWS threshold are labeled. Gene labels are not drawn to scale.

### Height

For breed–average height, we identified 27 SNPs above the SWS threshold and 18 above the GWS threshold across all individuals (Fig. 5a). The strongest association was observed for a SNP on chromosome 24 (position 22647289; $p\;=3.46\times \;10^{-6}$), located within the gene *SNTA1*. Additional genes with multiple SNPs surpassing the SWS threshold include *CERS3* (seven SNPs) on chromosome 3, *CBFA2T2* (16 SNPs) on chromosome 24, and *SNTA1* (four SNPs) on chromosome 24 (Supplementary Table 15). Single nucleotide polymorphisms within these three genes also reached the GWS threshold.

Next, we partitioned by body size. For small individuals, 34 SNPs were above the SWS threshold and 17 were above the GWS threshold for height (Fig. 5b). The strongest association was observed for a SNP on chromosome 9 (position 45711401; $p=1.44\times \;10^{-6}$), corresponding to the gene *PITPNA*. Within the small subgroup, additional variants above the SWS threshold were identified across multiple loci: on chromosome 3, *CERS3* (three SNPs), *IGF1R* (one SNP), *LRRC28* (one SNP), and *TTC23* (three SNPs); on chromosome 9, *PITPNA* (four SNPs), *SLC43A2* (two SNPs), *VPS53* (two SNPs), and *RPH3AL* (four SNPs); on chromosome 18, *HGF* (two SNPs); on chromosome 22, *COMMD6* (one SNP); and on chromosome 24, *CBFA2T2* (seven SNPs) and *SNTA1* (four SNPs) (Supplementary Table 15). Among these, SNPs in *TTC23*, *PITPNA*, *SLC43A2*, *RPH3AL*, CBFA2T2, and SNTA1 also reached the GWS threshold. For large individuals, a single SNP on chromosome 11 (position 9943557), associated with *PRR16*, was above the SWS threshold for height (Fig. 6c). A complete list of genes associated with height is provided in Supplementary Table 15.

### Weight

For breed–average weight, 66 SNPs were above the SWS threshold and 45 above the GWS threshold across all individuals (Fig. 6a). Nearly half of the SNPs above the SWS threshold (30 SNPs) were located within genes on chromosome 24. The strongest association was observed for a SNP on chromosome 32 (position 11413601; *P* = $3.24\times \;10^{-7}$), located within *PKD2*. Across all samples, additional SNPs above the SWS threshold were identified in several genes: on chromosome 3, *CERS3* (seven SNPs); on chromosome 20, *ZXDC* (three SNPs) and *TXNRD3* (one SNP); on chromosome 24, *SNTA1* (five SNPs), *CBFA2T2* (19 SNPs), *ZNF341* (two SNPs), and *CHMP4B* (four SNPs); on chromosome 32, *SEPT11* (one SNP), *CCNI* (one SNP), *PKD2* (11 SNPs), *SGMS2* (four SNPs), and *CYP2U1* (three SNPs); and on chromosome 35, *SPIDR* (five SNPs) (Supplementary Table 16). Genes on chromosomes 3, 20, 24, and 35 contained SNPs that also reached the GWS threshold, whereas *PKD2* was the only gene on chromosome 32 with SNPs above this threshold.

For small individuals, 31 SNPs were above the SWS threshold and 19 above the GWS threshold (Fig. 6b). Nearly one-third of the SNPs above the SWS threshold (20 SNPs) were located on chromosome 24. The strongest association was observed for a SNP on chromosome 24 (position 22660667; $\;p\;=5.88\times \;10^{-7}$), linked to the gene *SNTA1* (Fig. 6b). Additional SNPs above the SWS threshold were associated with genes including *CERS3* (one SNP) on chromosome 3; *RPH3AL* (four SNPs), PITPNA (four SNPs), and SLC43A2 (two SNPs) on chromosome 9; and *ADAM33* (two SNPs), *SIGLEC1* (two SNPs), *HSPA12B* (one SNP), *SNTA1* (four SNPs), *CBFA2T2* (nine SNPs), *ZNF341* (one SNP), and *CHMP4B* (one SNP) on chromosome 24 (Supplementary Table 16). Among these, SNPs in *PITPNA*, *SLC43A2*, *SNTA1*, *CBFA2T2*, and *ZNF341* also reached the GWS threshold.

In large individuals, 10 SNPs were above the SWS threshold and five were above the GWS threshold (Fig. 6c). The SNPs above the SWS threshold that also reached the GWS threshold were localized to two genes: *PKD2* (four SNPs) on chromosome 32 and *SPIDR* (six SNPs) on chromosome 35. A complete list of genes associated with weight is provided in Supplementary Table 16.

### Lifespan

For breed–average lifespan, 10 SNPs were above the SWS threshold and four were above the GWS threshold across all individuals (Fig. 7a). The strongest associated SNP was observed for a SNP on chromosome 20 (position 789204; $p=2.21\times \;10^{-5}$), located within the gene *TXNRD3*. Across all samples, additional SNPs above the SWS threshold were identified in several genes, including *ZXDC* (two SNPs) and *TXNRD3* (one SNP) on chromosome 20; *SEPT11* (one SNP) on chromosome 32; and *DRD3* (five SNPs) and *TIGIT* (one SNP) on chromosome 33 (Supplementary Table 10). All genes except *SEPT11* contained at least one SNP that reached the GWS threshold.

For small individuals, only a single gene, *RUNX2* (4 SNPs) on chromosome 12, was identified above the SWS threshold (Figure 7b and Supplementary Table 10). For large individuals, nine SNPs were above the SWS threshold, all of which reached the GWS threshold (Fig. 7c). The strongest association was observed for a SNP on chromosome 20 (position 789204; $p=1.17\times \;10^{-8}$), again within *TXNRD3.* All genes containing SNPs above the significance thresholds, including *TXNRD3* (one SNP), *ZXDC* (three SNPs), *CFAP100* (four SNPs), and *SLC41A3* (one SNP), were located on chromosome 20 (Supplementary Table 17). A complete list of genes associated with lifespan is provided in Supplementary Table 17.

## Discussion

Our study provides a comprehensive analysis of ROH across domesticated breed dogs and two non-breed dog populations, Chinese indigenous dogs and village dogs. Our results demonstrate how domestication and breed formation caused differences in patterns of genomic variation and may affect trait architecture. We show that ROH, quantified as the fraction of the genome contained within runs of homozygosity (F_ROH_), can be used to identify traits that are not fully additive. We focus on ROH because these genomic segments reflect recent demography and inbreeding (Thompson 2013; Purfield et al. 2017).

Previous work has shown that dogs originated from isolated wolf populations and strong artificial selection drove breed emergence (Boyko 2011; Freedman et al. 2016). Artificial selection reduced genetic diversity and increased homozygosity within breeds while maintaining substantial phenotypic variation between breeds (Boyko et al. 2010). In dogs, selection was often achieved through inbreeding, which also promoted the sharing of common genetic variants across individuals (Gregory 2009). As a result, breed establishment and standardization have led to relatively homogeneous genetic and phenotypic profiles within breeds. Introgression of desirable traits between breeds, together with inbreeding and reduced effective population size during breed formation, has contributed to shared haplotypes across breeds and distinct patterns of homozygosity within breeds (Parker et al. 2017).

Thus, in breed dogs, a substantial fraction of the genome is contained within long ROH due to the small number of individuals involved in breed establishment. These long ROH reflect recent parental relatedness and are closely tied to domestication and selective breeding practices. Despite high homozygosity, each breed exhibits distinct ROH patterns on average. As expected, breed dogs also carry a greater proportion of their genome in long ROH than non-breed populations, including Chinese indigenous dogs and village dogs (Supplementary Table 12). In contrast, these non-breed populations display lower levels of ROH, including reduced mean n_ROH_ and s_ROH_ (Supplementary Tables 12 and 13), reflecting higher genetic diversity and more outbred population histories. These results suggest that breed dogs share more recent common ancestry and greater background relatedness relative to village dogs and Chinese indigenous dogs (Boyko 2011), as well as the absence of strong bottlenecks associated with breed formation and more relaxed trait selection during population establishment (Yang et al. 2019; Mooney et al. 2021).

ROH hotspot analyses show that recombination rate explains some, but not all, of the variation in hotspot density across the genome (Supplementary Figs. 1 and 4). For example, on several chromosomes, ROH hotspots occur in regions with elevated recombination rates, indicating that additional factors contribute to hotspot retention. This is consistent with previous studies showing that recombination alone does not determine the distribution of ROH hotspot and coldspots (Pemberton and Szpiech 2018; Mooney et al. 2021). Our results suggest that ROH patterns are shaped by a combination of demography, artificial selection during breed formation, and inbreeding. Together, these processes generate distinct ROH patterns within breeds, shared regions among related breeds, and limited overlap between breed dogs and non-breed populations. In general, breeds within the same clade are more genetically similar than breeds from different clades, reflecting their more recent shared demographic history. Consequently, shared ROH hotspots across clades are relatively rare.

We also test whether there is a relationship between ROH and non-disease traits. We find a positive association between F_ROH_ and breed–average height and weight across all breed dogs and a negative association with lifespan (Fig. 4), even after correcting for covariates (Supplementary Table 8). The positive relationship with height contrasts with findings in human populations (McQuillan 2008; Joshi et al. 2015; Swinford et al. 2023) but may reflect the combined effects of artificial selection and inbreeding during breed formation, as body size has been a primary target of selection in dogs. When stratifying by weight, our results are consistent with previous studies on height in dogs (Hoopes et al. 2012). The negative association between F_ROH_ and lifespan suggests that increased inbreeding is linked to reduced lifespan, consistent with prior work in disease phenotypes in dogs (Mooney et al. 2021). When partitioning dogs by size, we observe a trend in which larger breeds have shorter lifespan (β=−0.15) and smaller breeds have longer lifespan (β=0.11), although these effects were not statistically significant (Supplementary Table 8) (Urfer et al. 2019). Previous studies also link body size with lifespan, highlighting this as an important area for future investigation (Galis et al. 2007). Including lifespan as a covariate in the height analysis reduced the effect size of F_ROH_ on height (from *β* ∼ 0.31 to *β* ∼ 0.19) and increased the *P*-value (p=0.034), indicating partial confounding between height and lifespan. Consistent with this, we observe a strong negative association between height and lifespan (β ∼ −0.83; $p=6.41\times \;10^{-41}$).

Our ROH-based GWAS identifies multiple loci associated with height, weight, and lifespan (Supplementary Tables 15 to 17). For height, three genes (*CERS3*, *CBFA2T2*, and *SNTA1*) exceed the GWS threshold across all individuals. All three genes were previously associated with human height (Kichaev et al. 2019; Yengo et al. 2022), and *CERS3* was also previously linked to body mass index (BMI) (Costa-Urrutia et al. 2019).

In small individuals, several genes (*PITPNA*, *LRRC28*, *TTC23*, *HGF*, *SLC43A2*, and *VPS53*) have also been implicated in human height, suggesting some sharing of genetic architecture underlying body size across species (Guo et al. 2018; Yengo et al. 2022). Further, we replicate a known signal at *IGF1R*, which exceeded the SWS threshold. Mutations in *IGF1R* have previously been linked to height in small dog breeds, such as Chihuahuas (Hoopes et al. 2012). We also identify *COMMD6* as a candidate gene associated with height in small individuals. Although *COMMD6* has primarily been studied in the context of immune function, our results suggest that it may also contribute to variation in height in dogs (COMMD6 gene information—The Human Protein Atlas 2024 Oct 30). Future work would be needed to determine its role. We also identify *RPH3AL* as a potential novel candidate. Although there is limited research linking this gene to height, its role in exocytosis and the secretion of growth hormones suggest a possible biological mechanism (RPH3AL gene information—The Human Protein Atlas 2024 Oct 30). In large individuals, the only gene above the SWS threshold was *PRR16*, which has also been associated with human height (Yengo et al. 2022).

The weight GWAS pinpoints *PKD2* as the strongest associated locus across all individuals. Functional studies in mouse models show that *PKD2* is involved in fat absorption and may contribute to obesity (Trujillo-Viera et al. 2021). Several additional genes in our study have been linked to weight-related phenotypes across species. For example, *SNTA1* was associated with live body weight (LBW) in goats, a trait commonly used to assess livestock health (Selionova et al. 2022). Another gene, *CBFA2T2*, regulates adipogenesis in both humans and mice and can influence obesity when its expression is altered (Ali et al. 2013; Matulewicz et al. 2017; Luo et al. 2020). *TXNRD3* influences adipocyte differentiation through its role in the Wnt signaling pathway, a key process in the regulation of body fat and energy storage (Kang et al. 2007; Kipp et al. 2012; Martínez-Montes et al. 2017). *CERS3*, which is associated with height, has been linked to BMI in humans (Costa-Urrutia et al. 2019). In addition, *SPIDR* and *ZNF341* are associated with total body fat and bone mineral content, respectively (Shapses and Sukumar 2012; Tachmazidou et al. 2013; Blake et al. 2021). *CHMP4B* was linked to human birth weight, although it has not been directly associated with obesity or body mass (Warrington et al. 2019; CHMP4B gene information—The Human Protein Atlas 2024 Oct 30). Similarly, *ZXDC* has primarily been studied in immune system regulation and cancer biology—its potential role on affecting weight could be an area for future research of *ZXDC*.

In small individuals, we observe overlapping associations between weight and *SNTA1*, *ZNF341*, and *CBFA2T2*, which may reflect shared haplotypes or underlying breed structure. We also identify candidates specific to this subgroup, including *PITPNA* (also associated with height) and *SLC43A2*, both of which have been shown to decrease body weight in knockout mouse models (Alb et al. 2003; Guetg et al. 2015). In large individuals, only *PKD2* and *SPIDR* were associated with weight, both of which have established roles in body fat regulation.

For lifespan, we identify several associations of interest across genes linked to survival and disease-related processes. Across all individuals, significant SNPs were seen within *TXNRD3*, *DRD3*, *TIGIT*, *SEPT11*, and *ZXDC*. *TXNRD3* has been linked to reduced survival in various cancers (Wu et al. 2021). Although direct evidence linking *TXNRD3* to lifespan is limited, members of the thioredoxin reductase family have been implicated in aging biology. For example, *TXNRD2* expression and activity are elevated in multiple long-lived species, and overexpression of *TXNRD2* was shown to extend lifespan in *Drosophila* (Pickering et al. 2017). Together, these findings suggest that redox regulation may influence lifespan and support *TXNRD3* as a biologically plausible candidate gene. *DRD3*, which encodes the D3 dopamine receptor, has been implicated in schizophrenia, a condition associated with decreased life expectancy (Hjorthøj et al. 2017; Kang et al. 2023). In addition, loss of D3 receptor function in mice leads to cardiac fibrosis and other aging-related phenotypes (Johnson et al. 2013), suggesting that *DRD3* may contribute to variation in lifespan. Similarly, *TIGIT* and *SEPT11* have been tied tumor progression and reduced lifespan (Johnston et al. 2014; Chauvin et al. 2015; Fu et al. 2023), while *ZXDC* has been associated with cervical cancer metastasis, which negatively impacts patient survival (Mao et al. 2023).

In small dogs, we observe an association with *RUNX2*, a gene previously linked to multiple cancers and poor prognosis (Zhao et al. 2021). In large individuals, *SLC41A3* was associated with lifespan and has been connected to liver hepatocellular carcinoma, a major contributor to cancer-related death (Chang et al. 2021). We also identify *CFAP100* as a candidate gene in this group, which has not previously been linked to lifespan.

Overall, our results demonstrate that quantitative traits in dogs are polygenic, with contributions from multiple loci, and that quantitative phenotypes may not be fully additive. We recover a more modest number of associations after correcting for inflation (50 for height, 84 for weight, and 21 for lifespan). In comparison to humans, 25,551 associations have been reported for height, 2,233 for weight, and 664 for lifespan (Sollis et al. 2023). This pattern is consistent with previous work suggesting that dogs exhibit a more simplified genetic architecture for complex traits relative to humans (Boyko et al. 2010; Freedman et al. 2016). Additionally, most associations were not shared across the groupings (eg all individuals, small dogs, and large dogs). We believe this reflects differences in genetic architecture and allele frequencies across size classes, as well as differences in power when stratifying individuals.

One of the advantages to the simplified trait architecture in dogs is that we have enough power to detect associations with relatively small sample sizes. Achieving comparable power in human GWAS studies requires sample sizes on the order of hundreds of thousands to millions of individuals (Kichaev et al. 2019; Yengo et al. 2022). In contrast, we were able to detect SNPs associated with these three quantitative traits using fewer than 1,000 dogs. This boost in power is once again a result of the unique demographic history of dogs, where domestication and breed formation bottlenecks simplified architecture to consist of fewer variants with larger effect (Boyko et al. 2010).

Importantly, we validate our ROH-based GWAS by attempting to replicate a previously identified peak on chromosome 13 within *RSPO2*, linked to furnish in dogs (Plassais et al. 2019). We identify the same peak (Supplementary Fig. 26), although it did not reach genome-wide or SWS after correction (Supplementary Fig. 27). In addition to this signal, we observe several novel peaks not reported in previous studies. Replication of this peak supports the validity of our approach and indicates that ROH-based GWAS captures complementary information relative to standard SNP-based methods. This is because it leverages extended regions of homozygosity to detect signals that may reflect IBD and recessive effects that are not captured by single-variant, typically additive models. Further, we were also able to find novel loci not recovered in previous SNP-based studies (Hayward et al. 2016; Plassais et al. 2019) by testing with ROH. This increased detection power highlights the potential of ROH-based approaches to improve GWAS, especially in the case of non-additivity. By leveraging shared ancestry and linkage among variants, haplotype-based methods provide an alternative framework for detecting genotype–phenotype associations. More broadly, such approaches may be particularly useful in domesticated species, managed populations, and natural populations experiencing bottlenecks or strong selection. Newly identified genes from this study should be interpreted as candidate regions, represent promising targets for future functional investigation, and highlight additional avenues for exploring complex trait architecture and non-additive effects in domesticated species.

Several limitations of this study should be noted. First, phenotypic values were based on breed averages, restricting inference to between-breed associations and limiting our ability to detect within-breed variation. Although this approach has been validated in previous studies (Jones et al. 2008; Boyko et al. 2010; Rimbault et al. 2013; Hayward et al. 2016; Plassais et al. 2017, 2019), it assumes minimal within-breed variance. This assumption is supported by breed standards established by organizations such as the AKC, which use phenotypic uniformity within breeds. We further demonstrate that when within-breed variance is low, effect size estimates derived from breed–average phenotypes closely approximate those obtained from individual-level data (Supplementary Fig. 31 and Appendix). Nevertheless, breed–average analyses may introduce bias if phenotypic differences among breeds are correlated with demography or inbreeding. To mitigate this concern, we accounted for relatedness using an ROH-based kinship matrix within a mixed-model framework. While some residual confounding due to population structure may remain, the persistence of associations after correction suggests that population structure alone is unlikely to explain the observed patterns. Second, breed assignments based on neighbor-joining trees and AKC categories may not fully capture the complex evolutionary histories of some breeds, particularly those with mixed ancestry, potentially introducing uncertainty into breed–group classifications. Third, we observed a concentration of association signals on smaller chromosomes (Supplementary Fig. 8; Supplementary Table 18), so we cannot exclude the possibility that linkage has introduced some biases. In addition, there is elevated GC content on certain chromosomes such as chromosome 32 in the CanFam3.1 assembly (Wang et al. 2021), which could introduce noise into our results. Despite these limitations, the consistent associations between F_ROH_ and multiple traits support a role for domestication, breed formation, and inbreeding in shaping complex, non-disease traits in domestic dogs.

In summary, our work advances understanding the genetic architecture underlying quantitative traits in dogs. We show that inbreeding, as measured by ROH, occurred alongside selection for extreme phenotypes during breed formation, leaving distinct signatures across the canine genome. ROH are associated with complex, non-disease phenotypes and provide insights into how domestication, selective breeding, and demographic history have shaped trait variation in dogs.

## Supplementary Material

jkag195_Supplementary_Data

## Data Availability

All code is available at https://github.com/jaam92/QuantTraitInDogs, and all data have been published. Whole-genome sequence data is available on NCBI, accession number: PRJNA448733. The source phenotype data was obtained from (Plassais et al. 2019). All summary data are contained within the article and the supplementary information. Supplemental material available at G3 online.
