# Supplementary material for: Genotypic and phenotypic consequences of domestication in dogs
Source: G3 (Bethesda). 2026 Jul 17;16(9):jkag195. doi: 10.1093/g3journal/jkag195 (PMC13535439; doi:10.1093/g3journal/jkag195)
Supplement: Supplementary file 1 [file jkag195_supplementary_data.zip › Supplemental_Figures_and_Text_G3-2026-406887.pdf]

Supplementary Figures

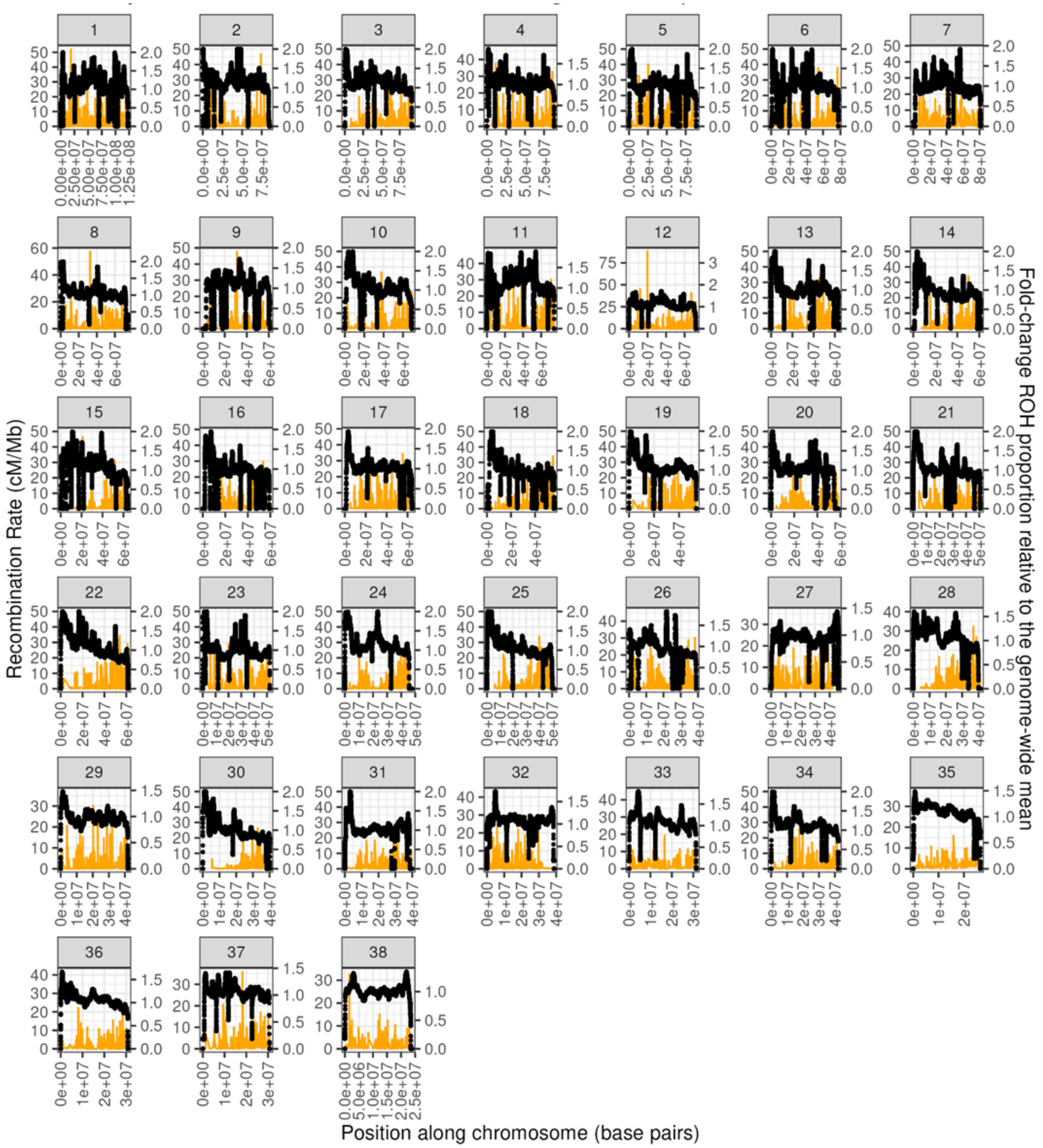

Figure S1. Genome-wide comparison of recombination rate and ROH enrichment across autosomes. *Each panel corresponds to an autosomal chromosome (1-38), with genomic position (base pairs) on the x-axis. The left y-axis shows recombination rate (cM/Mb; black line), while the right y-axis shows the fold-change in ROH proportion relative to the genome-wide mean (orange bars). Patterns highlight the relationship between local recombination rate variation and relative enrichment or depletion of ROH across the genome.*

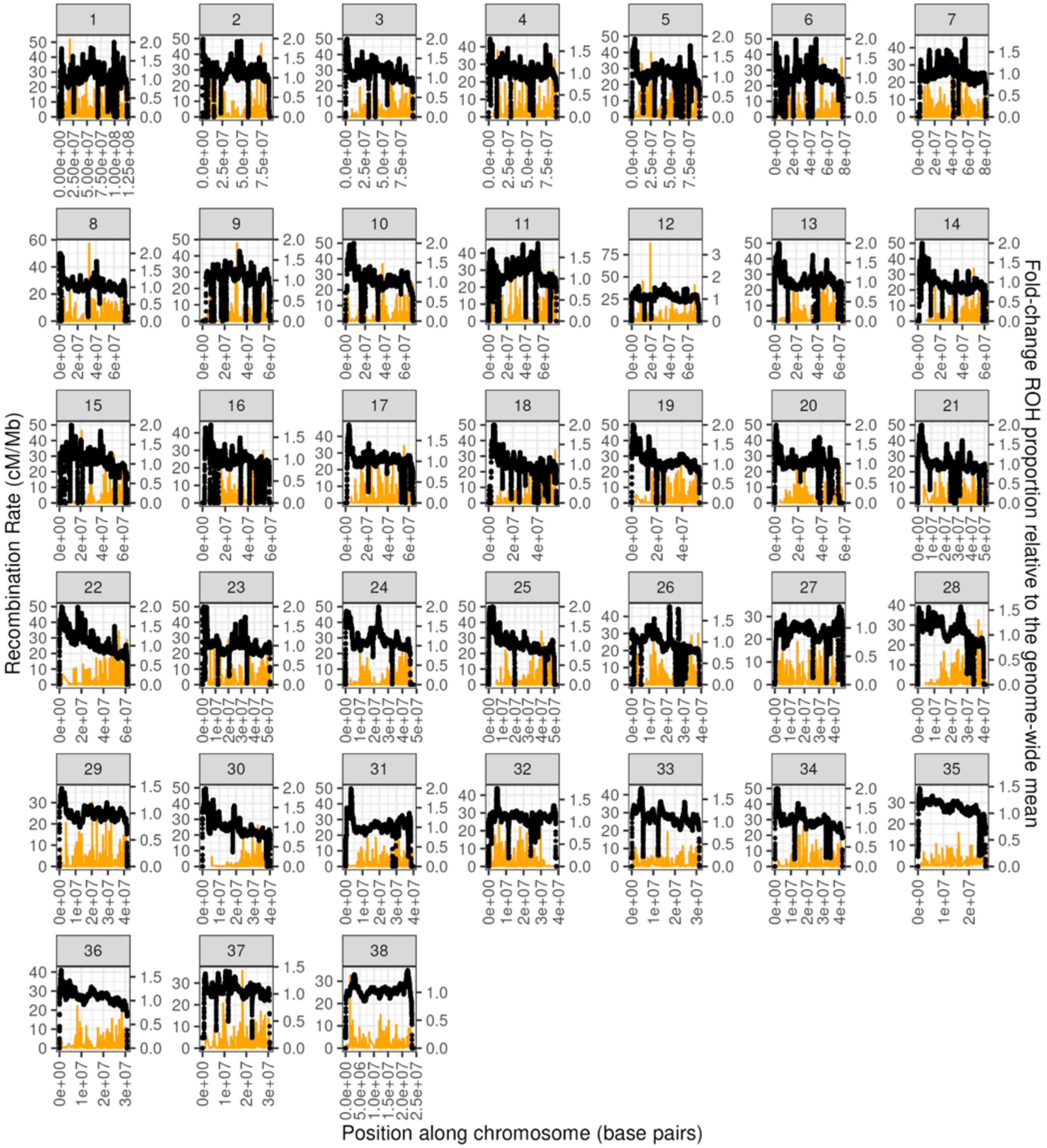

Figure S2. Genome-wide comparison of recombination rate and ROH enrichment across autosomes in breed dogs only (excluding Chinese Indigenous and Village Dogs). *Each panel corresponds to an autosomal chromosome (1-38), with genomic position (base pairs) on the x-axis. The left y-axis shows recombination rate (cM/Mb; black line), while the right y-axis shows the fold-change in ROH proportion relative to the genome-wide mean (orange bars). Patterns highlight the relationship between local recombination rate variation and relative enrichment or depletion of ROH across the genome.*

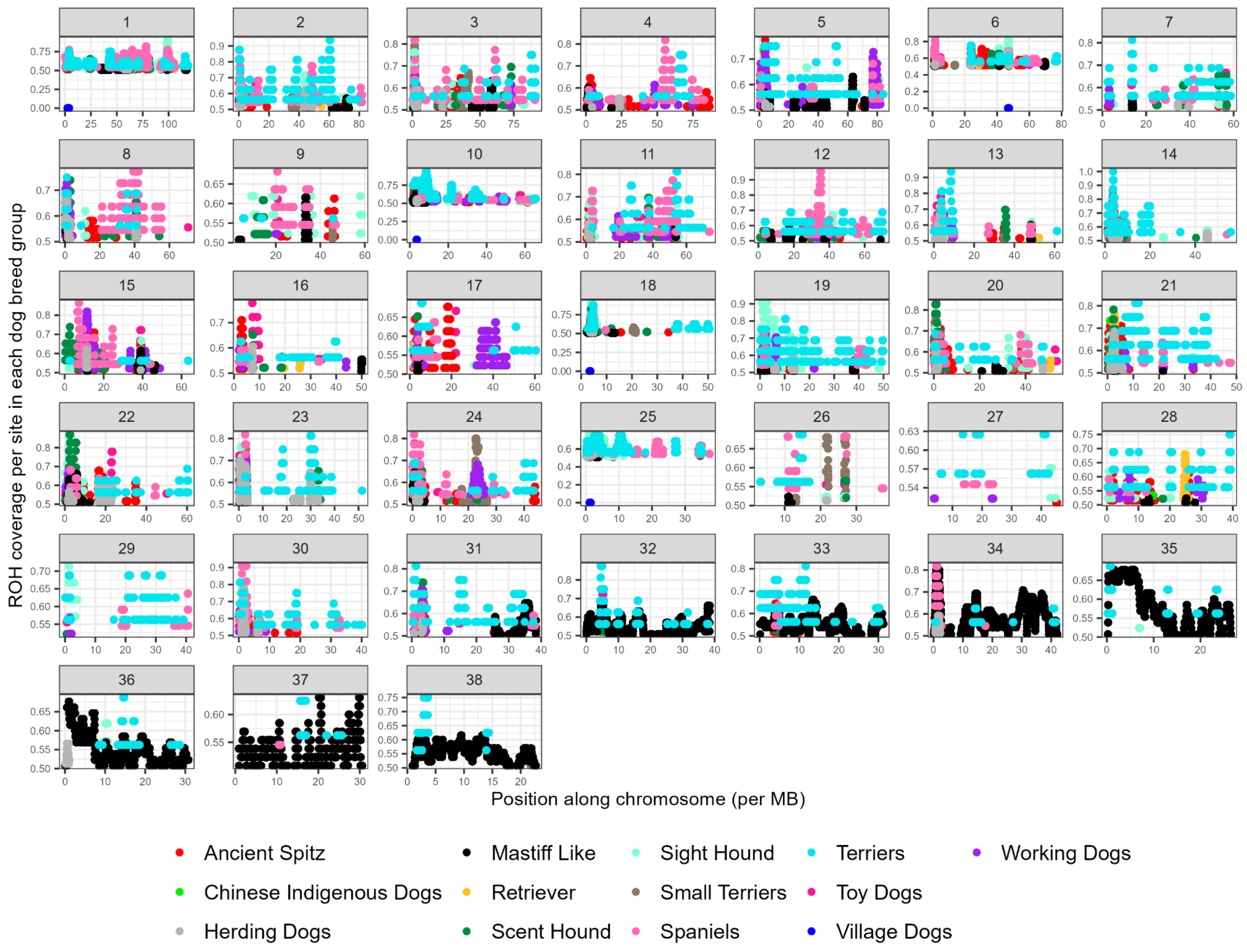

Figure S3. ROH coverage per site across autosomes for each dog breed group. *Each panel corresponds to an autosomal chromosome (1-38), with genomic position (Mb) on the x-axis. The y-axis represents the proportion of individuals per breed group with a given site in an ROH (coverage >0.5). Points are colored by breed group.*

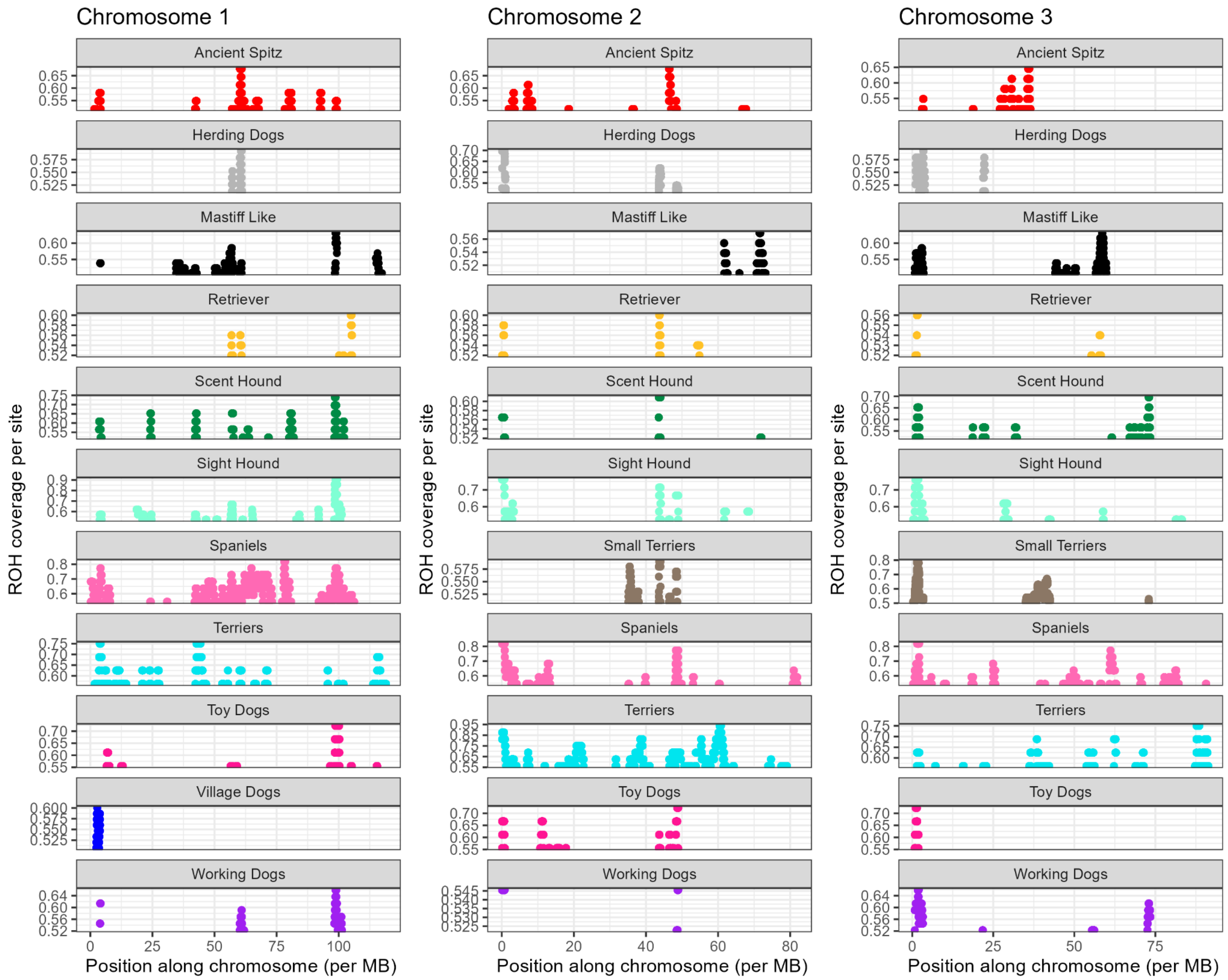

Figure S4. ROH coverage per site across chromosomes 1-3 for each dog breed group. *Within each chromosome, panels are stratified by breed group. The x-axis shows genomic position (Mb), and the y-axis shows the proportion of individuals per breed group with a given site in an ROH(coverage >0.5).*

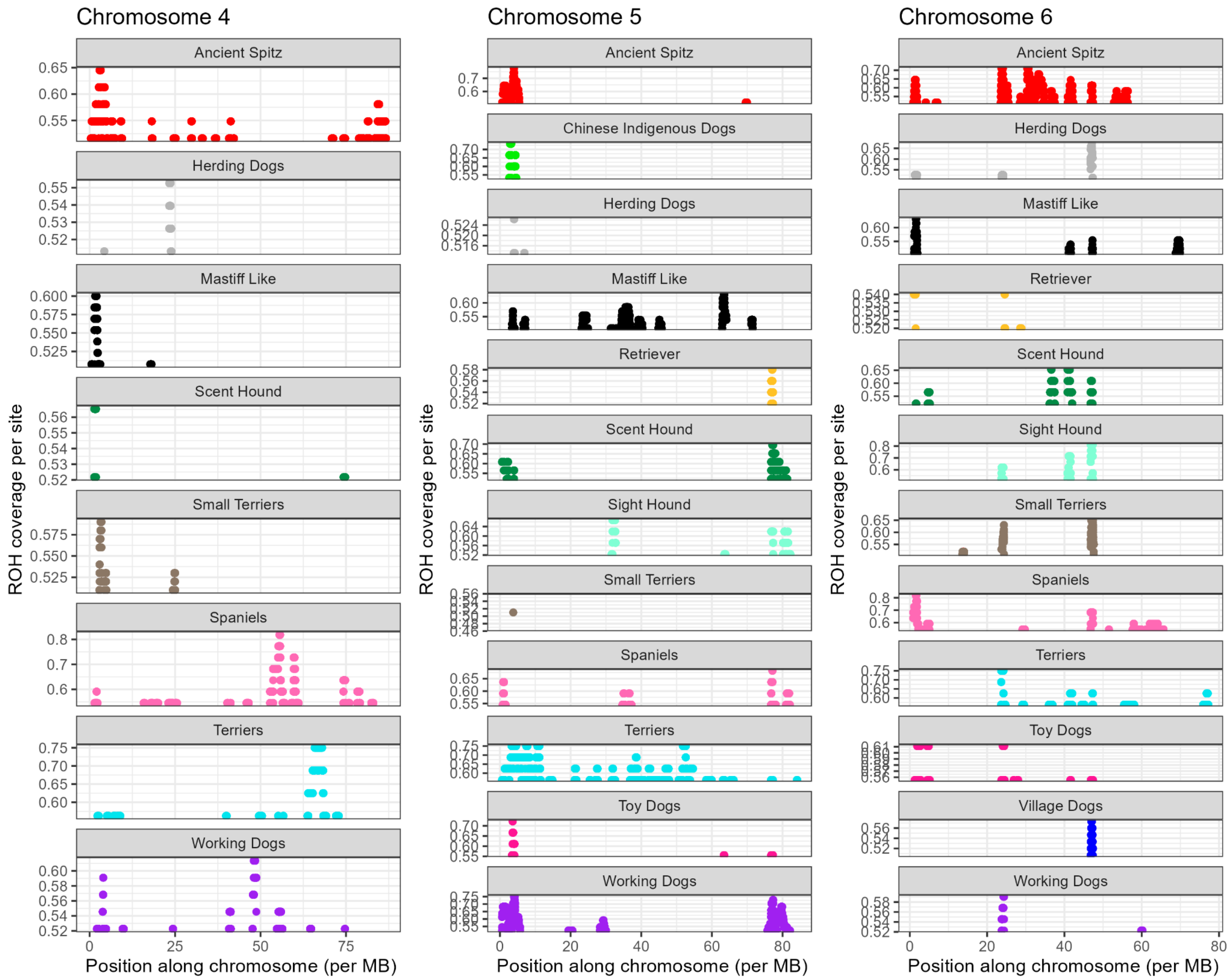

Figure S5. ROH coverage per site across chromosomes 4-6 for each dog breed group. *Within each chromosome, panels are stratified by breed group. The x-axis shows genomic position (Mb), and the y-axis shows the proportion of individuals per breed group with a given site in an ROH(coverage >0.5).*

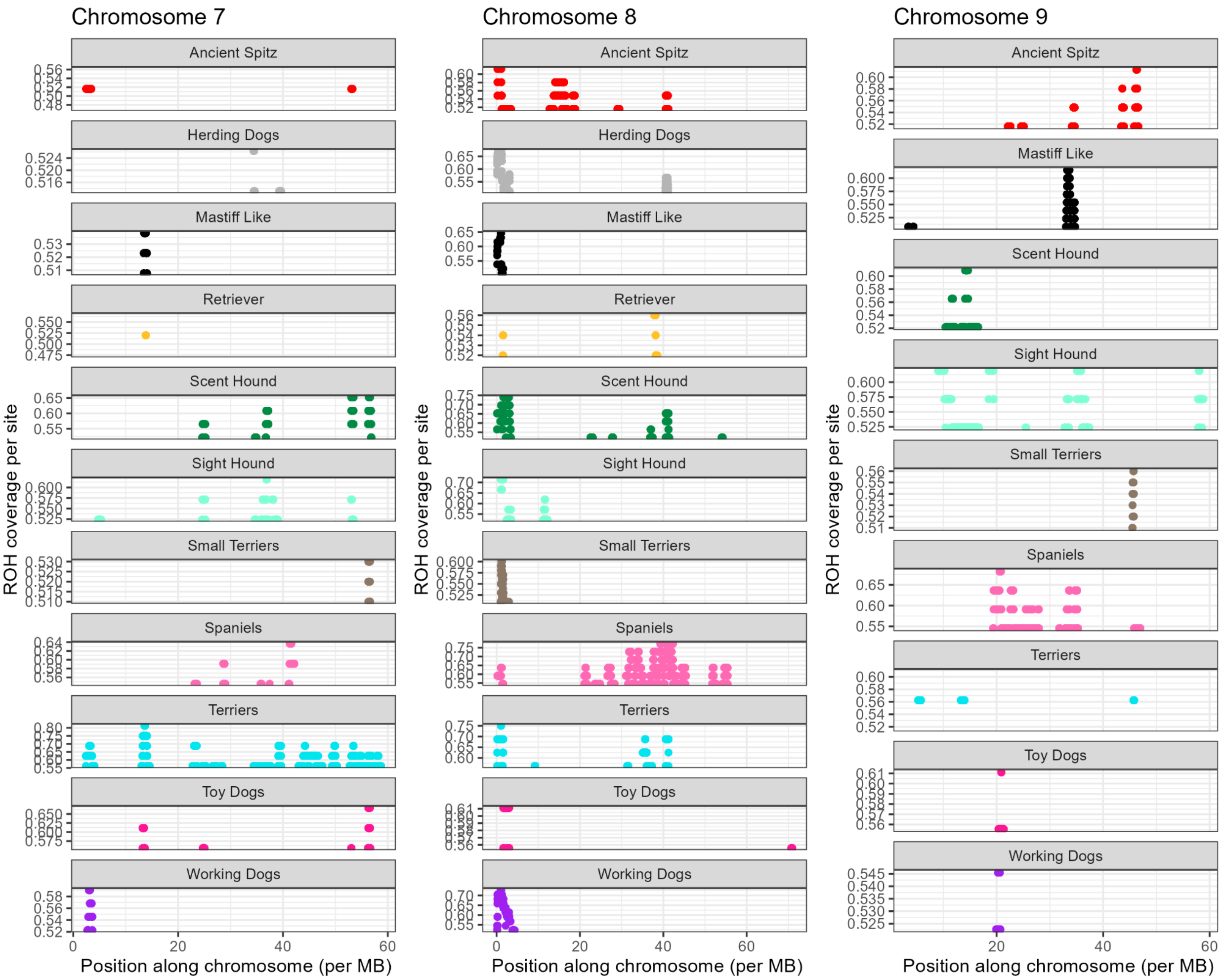

Figure S6. ROH coverage per site across chromosomes 7-9 for each dog breed group. *Within each chromosome, panels are stratified by breed group. The x-axis shows genomic position (Mb), and the y-axis shows the proportion of individuals per breed group with a given site in an ROH(coverage >0.5).*

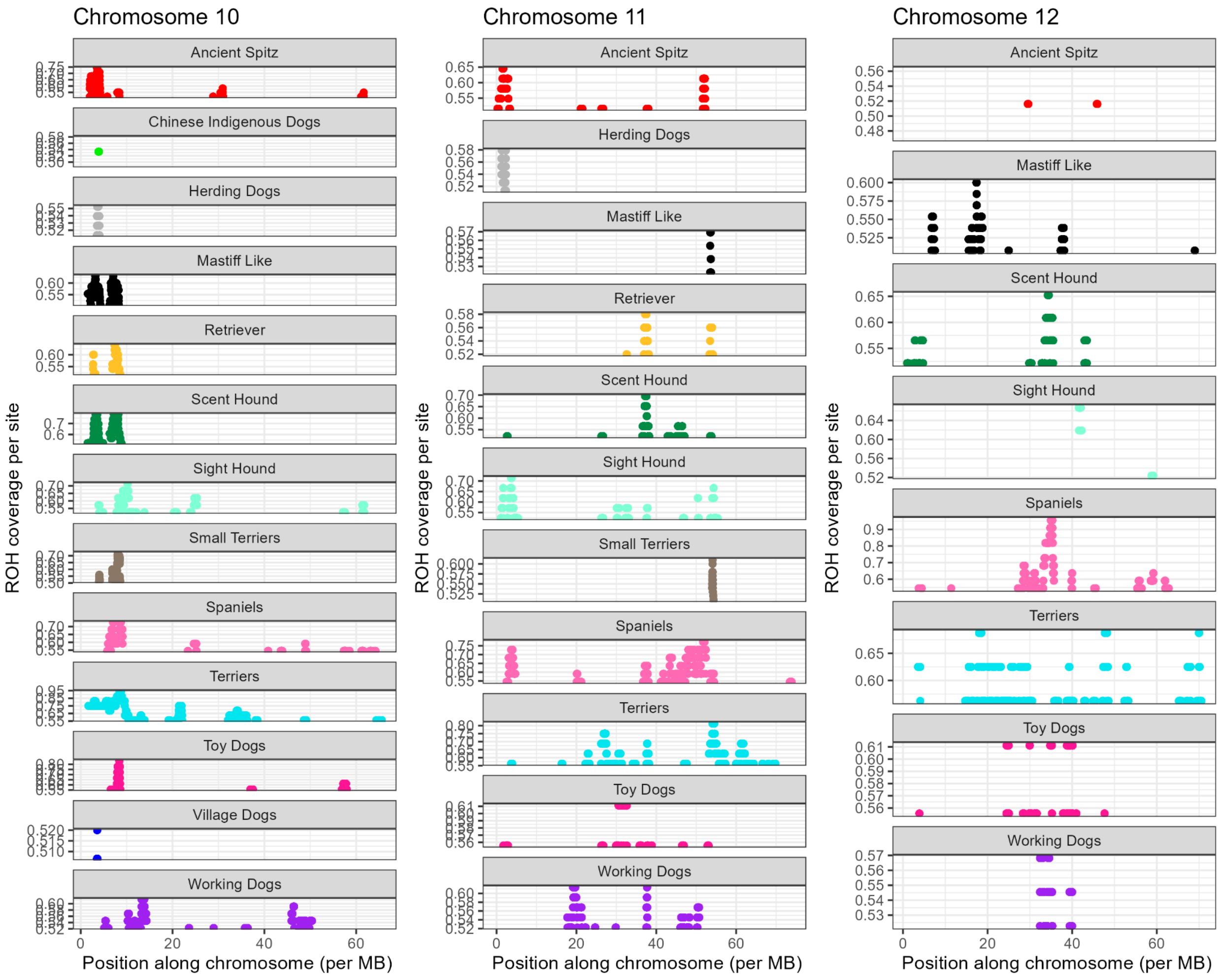

Figure S7. ROH coverage per site across chromosomes 10-12 for each dog breed group. *Within each chromosome, panels are stratified by breed group. The x-axis shows genomic position (Mb), and the y-axis shows the proportion of individuals per breed group with a given site in an ROH(coverage >0.5).*

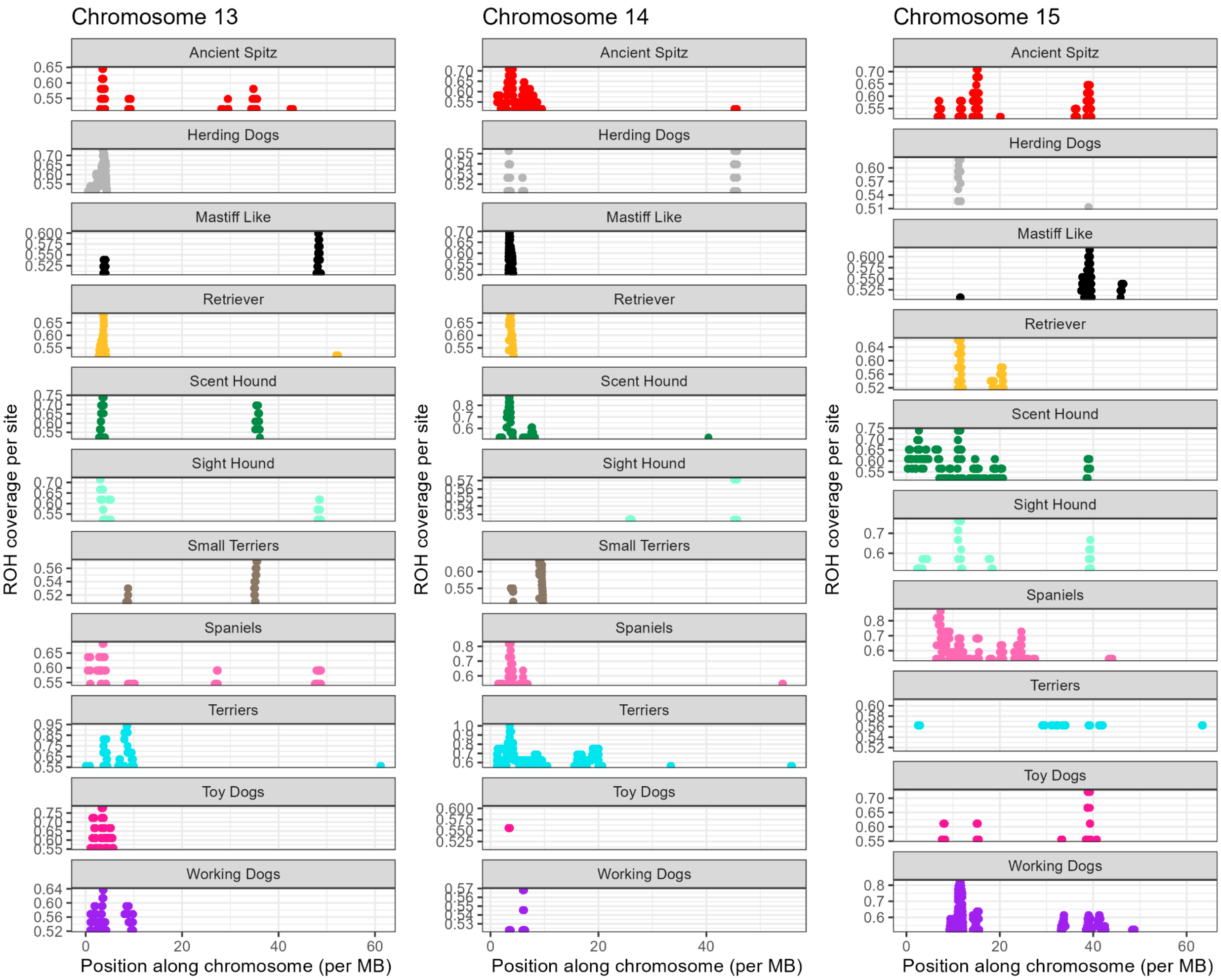

Figure S8. ROH coverage per site across chromosomes 13-15 for each dog breed group. *Within each chromosome, panels are stratified by breed group. The x-axis shows genomic position (Mb), and the y-axis shows the proportion of individuals per breed group with a given site in an ROH(coverage >0.5).*

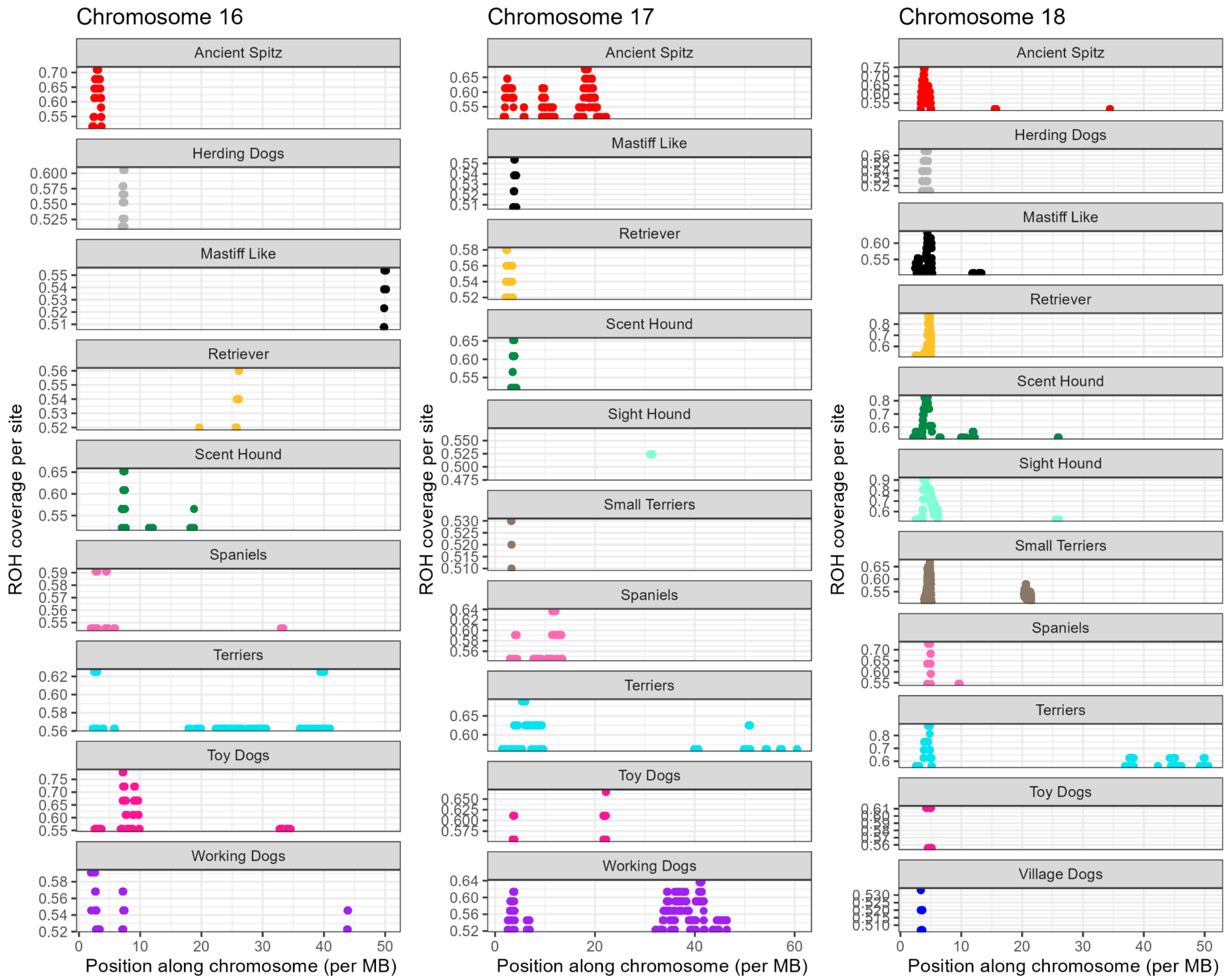

Figure S9. ROH coverage per site across chromosomes 16-18 for each dog breed group. *Within each chromosome, panels are stratified by breed group. The x-axis shows genomic position (Mb), and the y-axis shows the proportion of individuals per breed group with a given site in an ROH(coverage >0.5).*

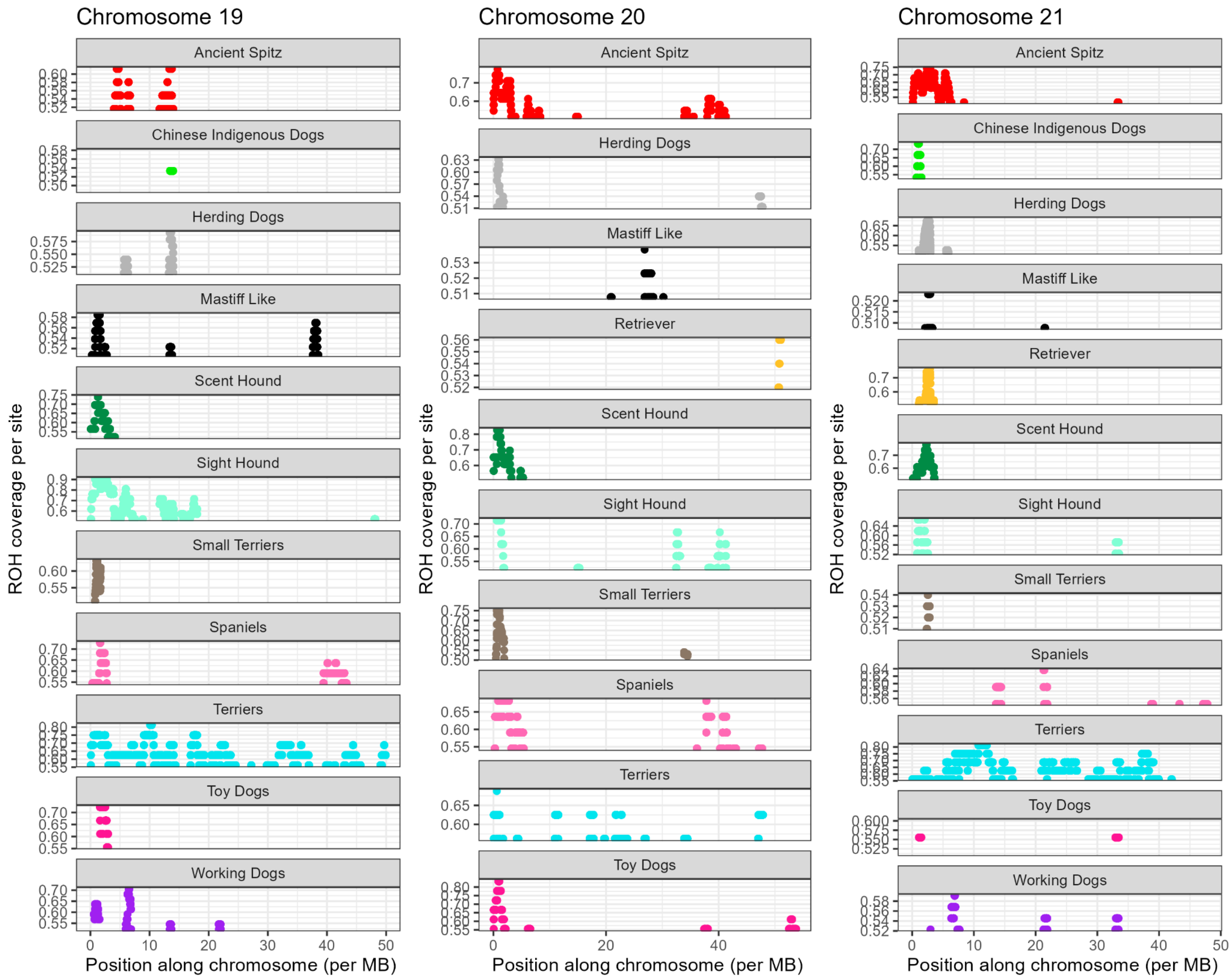

Figure S10. ROH coverage per site across chromosomes 19-21 for each dog breed group. *Within each chromosome, panels are stratified by breed group. The x-axis shows genomic position (Mb), and the y-axis shows the proportion of individuals per breed group with a given site in an ROH(coverage >0.5).*

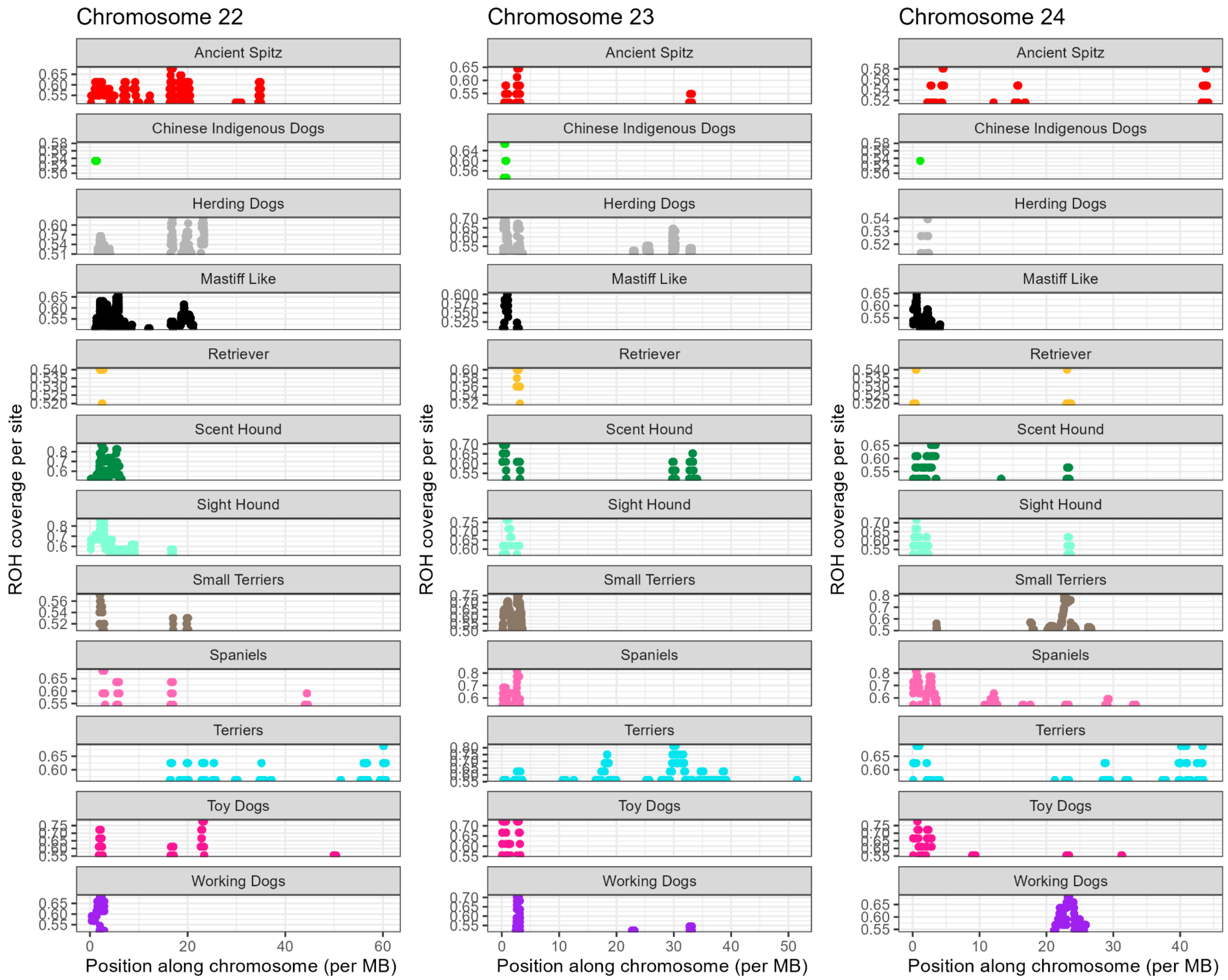

Figure S11. ROH coverage per site across chromosomes 22-24 for each dog breed group. *Within each chromosome, panels are stratified by breed group. The x-axis shows genomic position (Mb), and the y-axis shows the proportion of individuals per breed group with a given site in an ROH(coverage >0.5).*

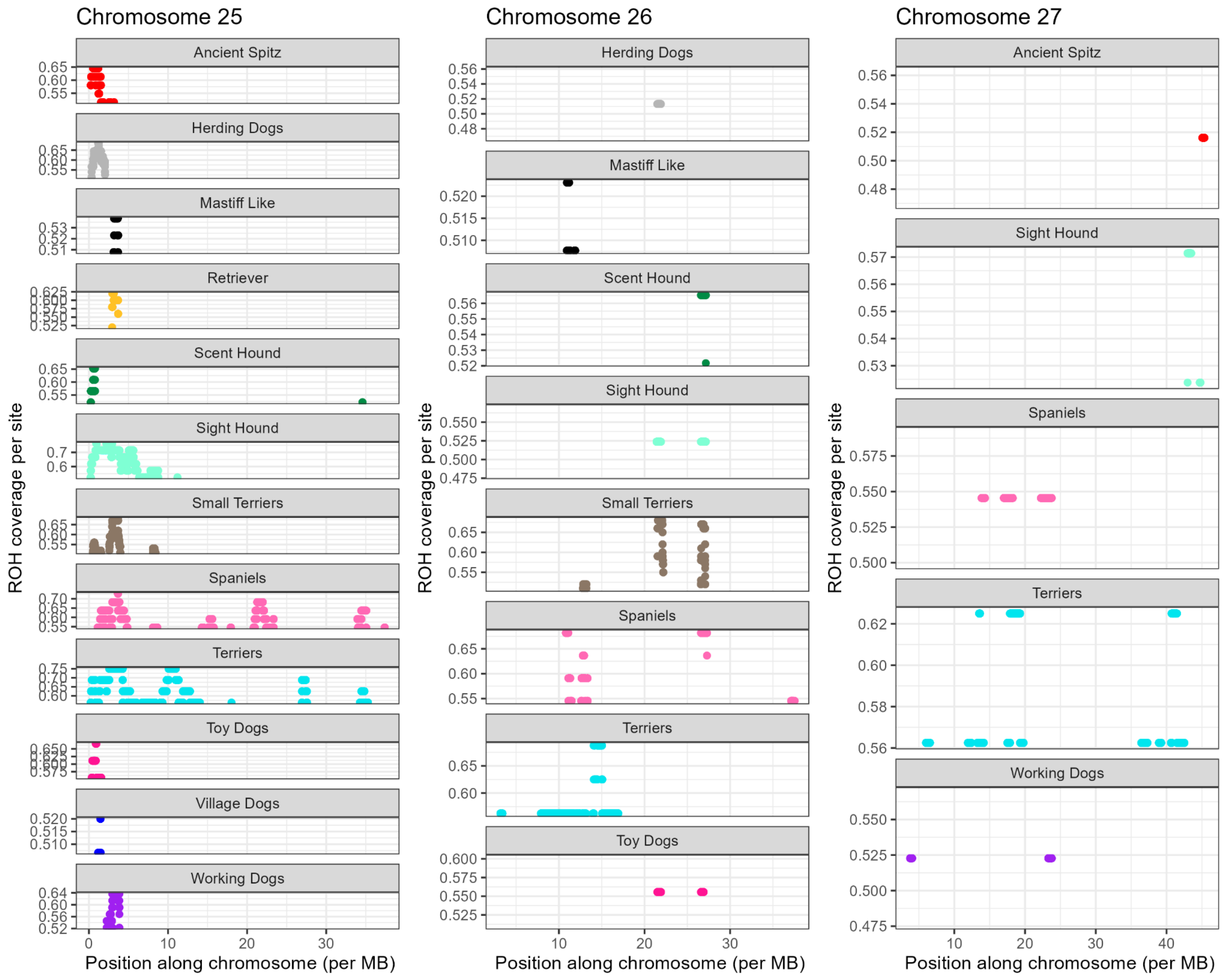

Figure S12. ROH coverage per site across chromosomes 25-27 for each dog breed group. *Within each chromosome, panels are stratified by breed group. The x-axis shows genomic position (Mb), and the y-axis shows the proportion of individuals per breed group with a given site in an ROH(coverage >0.5).*

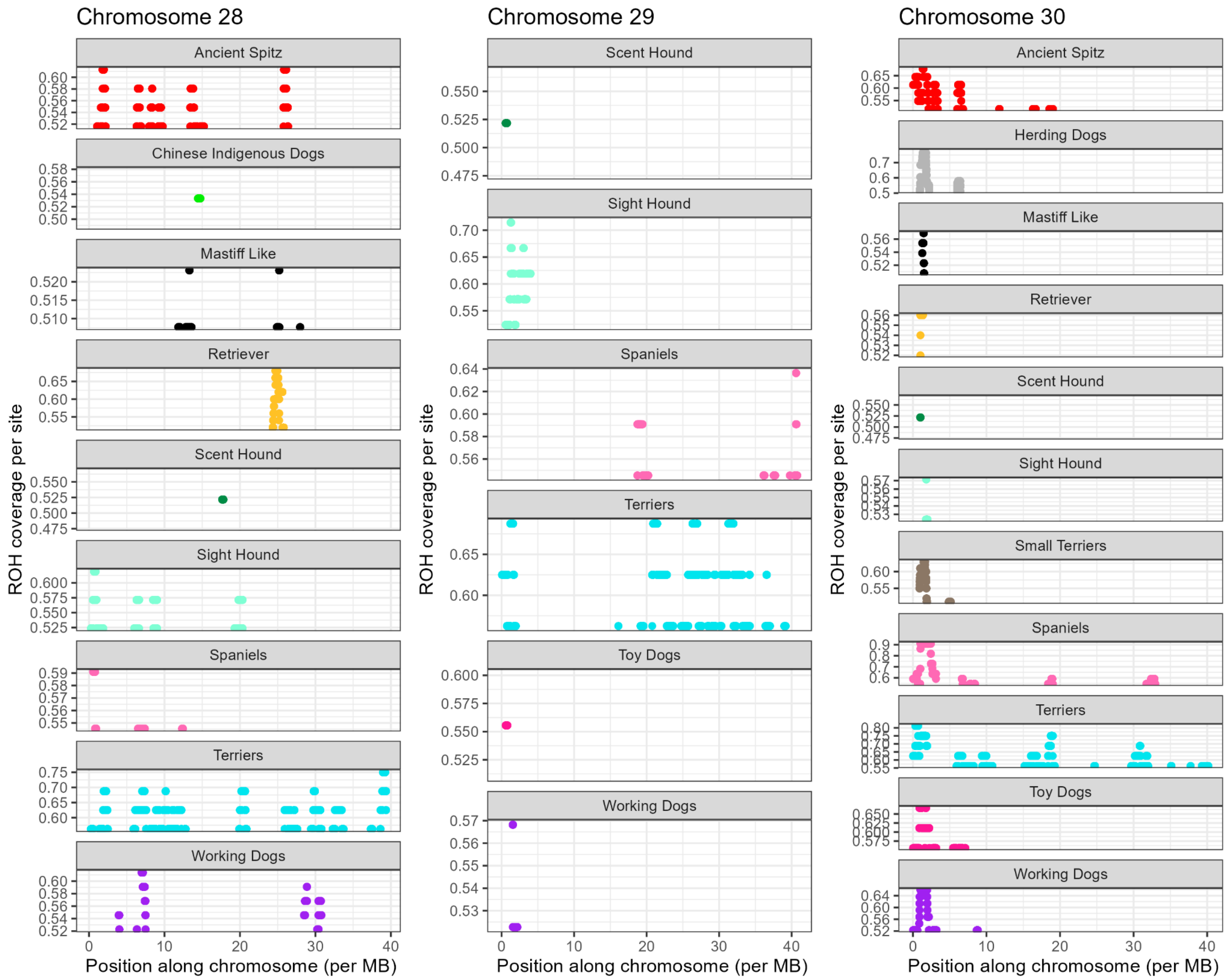

Figure S13. ROH coverage per site across chromosomes 28-30 for each dog breed group. *Within each chromosome, panels are stratified by breed group. The x-axis shows genomic position (Mb), and the y-axis shows the proportion of individuals per breed group with a given site in an ROH(coverage >0.5).*

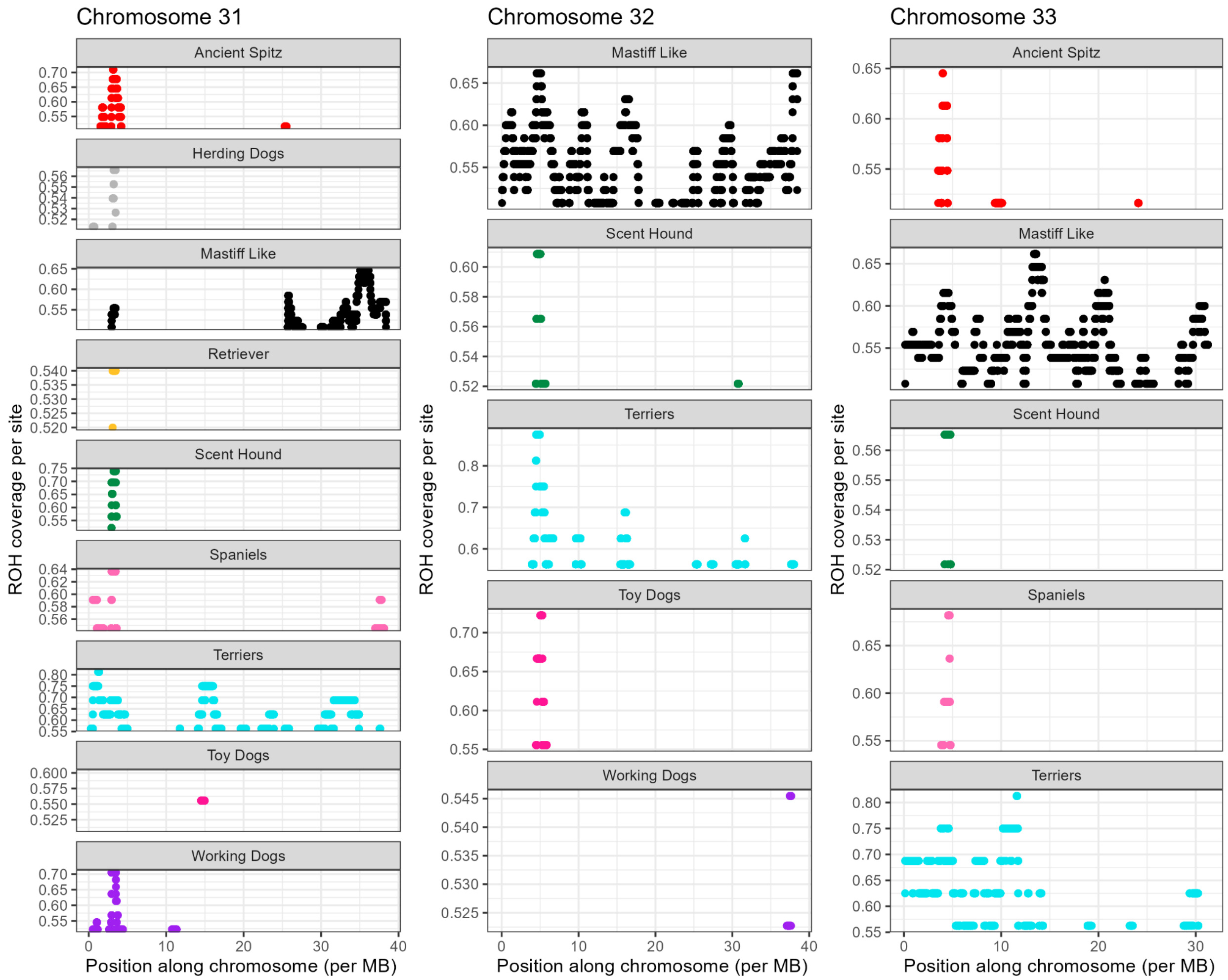

Figure S14. ROH coverage per site across chromosomes 31-33 for each dog breed group. *Within each chromosome, panels are stratified by breed group. The x-axis shows genomic position (Mb), and the y-axis shows the proportion of individuals per breed group with a given site in an ROH(coverage >0.5).*

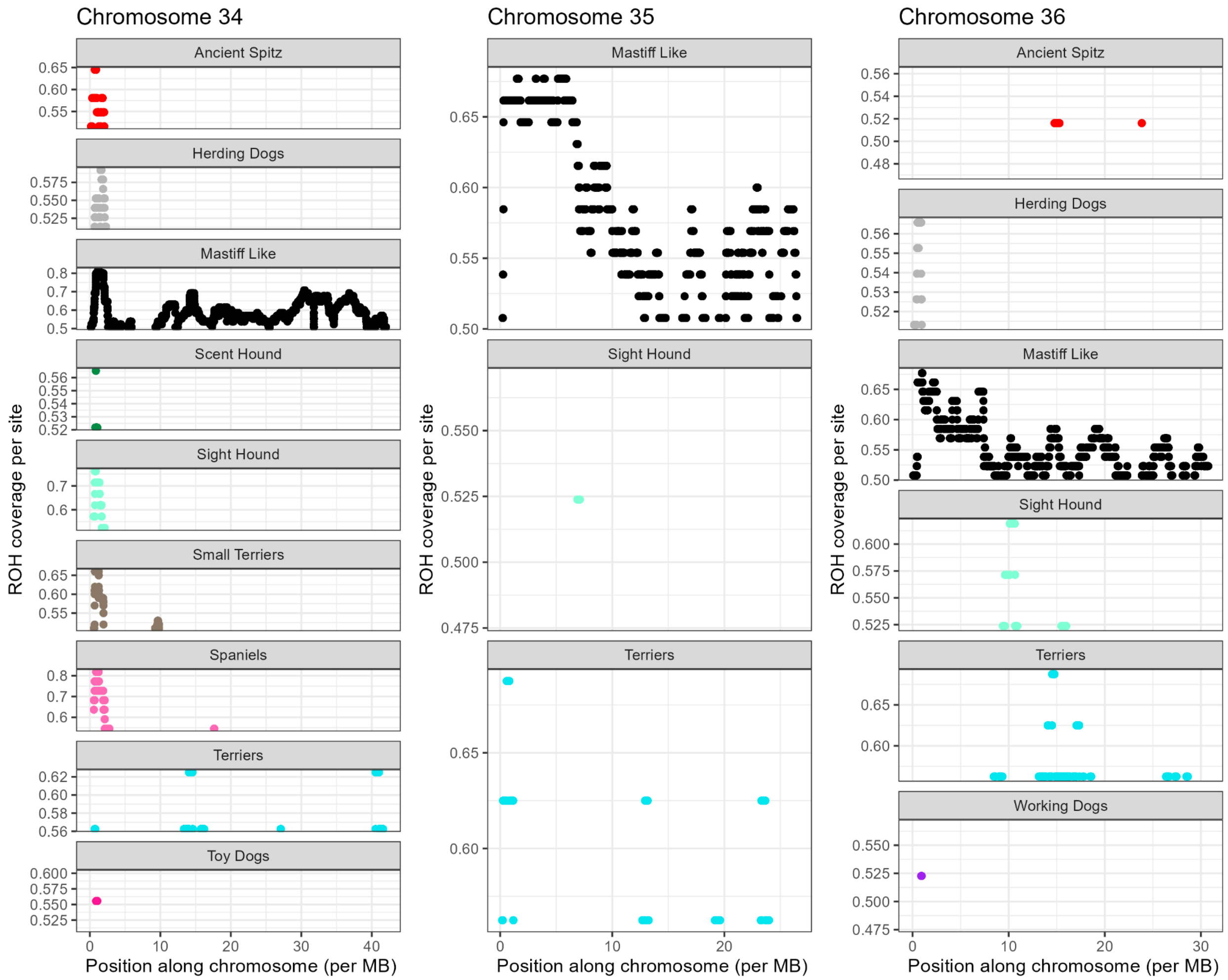

Figure S15. ROH coverage per site across chromosomes 34-36 for each dog breed group. *Within each chromosome, panels are stratified by breed group. The x-axis shows genomic position (Mb), and the y-axis shows the proportion of individuals per breed group with a given site in an ROH(coverage >0.5).*

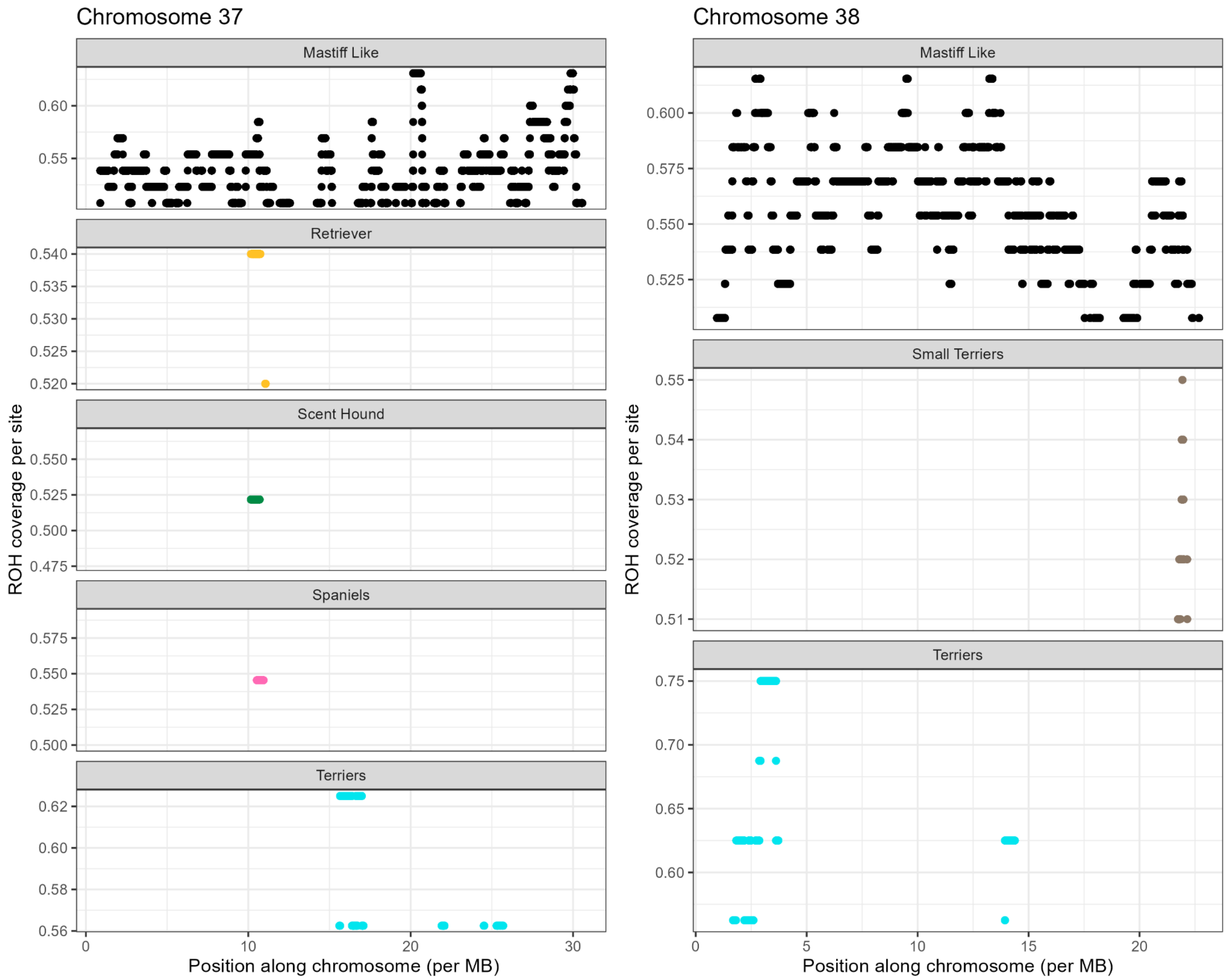

Figure S16. ROH coverage per site across chromosomes 37 and 38 for each dog breed group. *Within each chromosome, panels are stratified by breed group. The x-axis shows genomic position (Mb), and the y-axis shows the proportion of individuals per breed group with a given site in an ROH(coverage >0.5).*

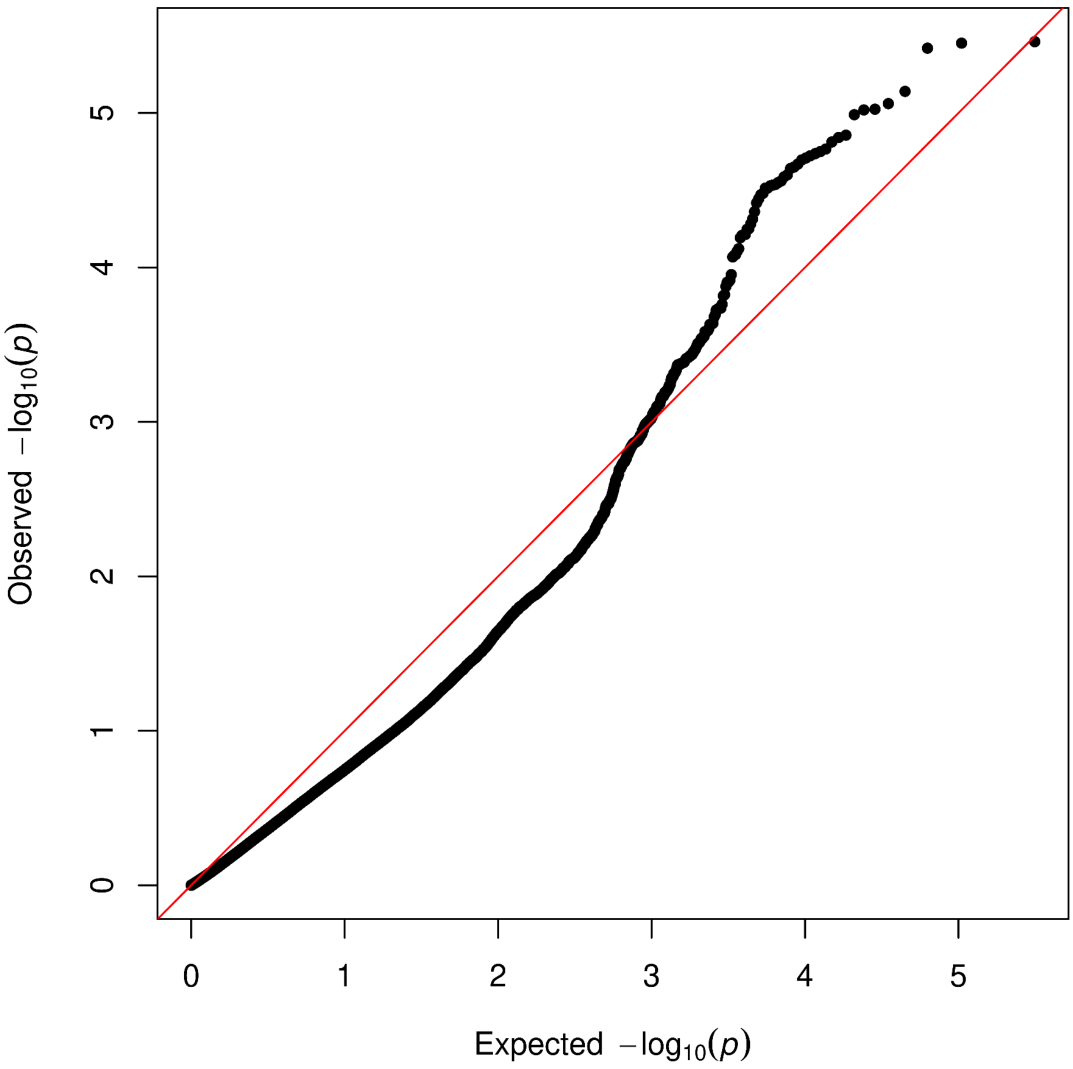

Figure S17. Quantile-quantile (Q-Q) plot comparing observed and expected −log₁₀(p) values from the GLMM-based GWAS for body height (all individuals). *The red diagonal line represents the null expectation under no association; deviations from this line indicate inflation or true association signals.*

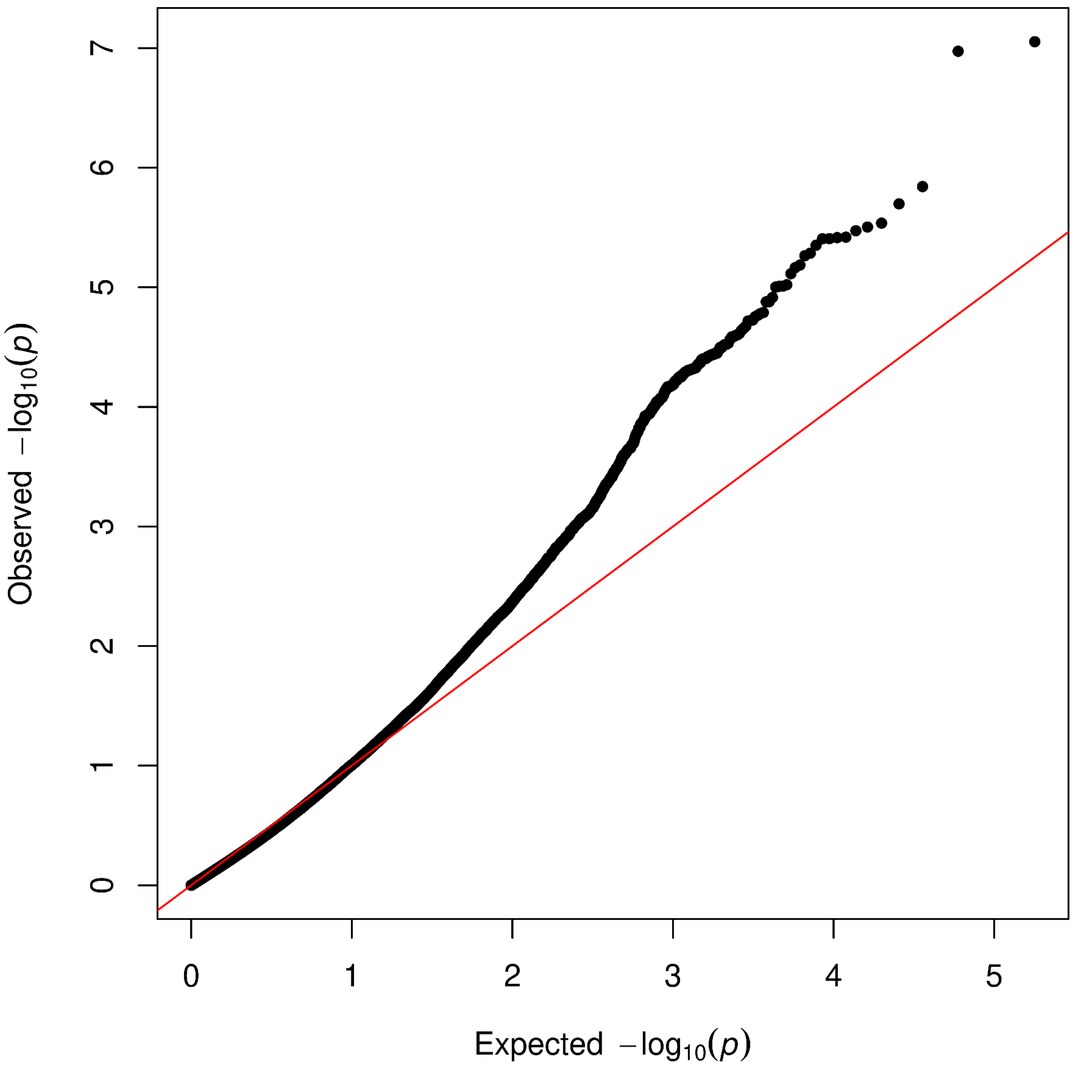

Figure S18. Quantile-quantile (Q-Q) plot comparing observed and expected $-\log_{10}$(p) values from the GLMM-based GWAS for body height (small individuals). *The red diagonal line represents the null expectation under no association; deviations from this line indicate inflation or true association signals.*

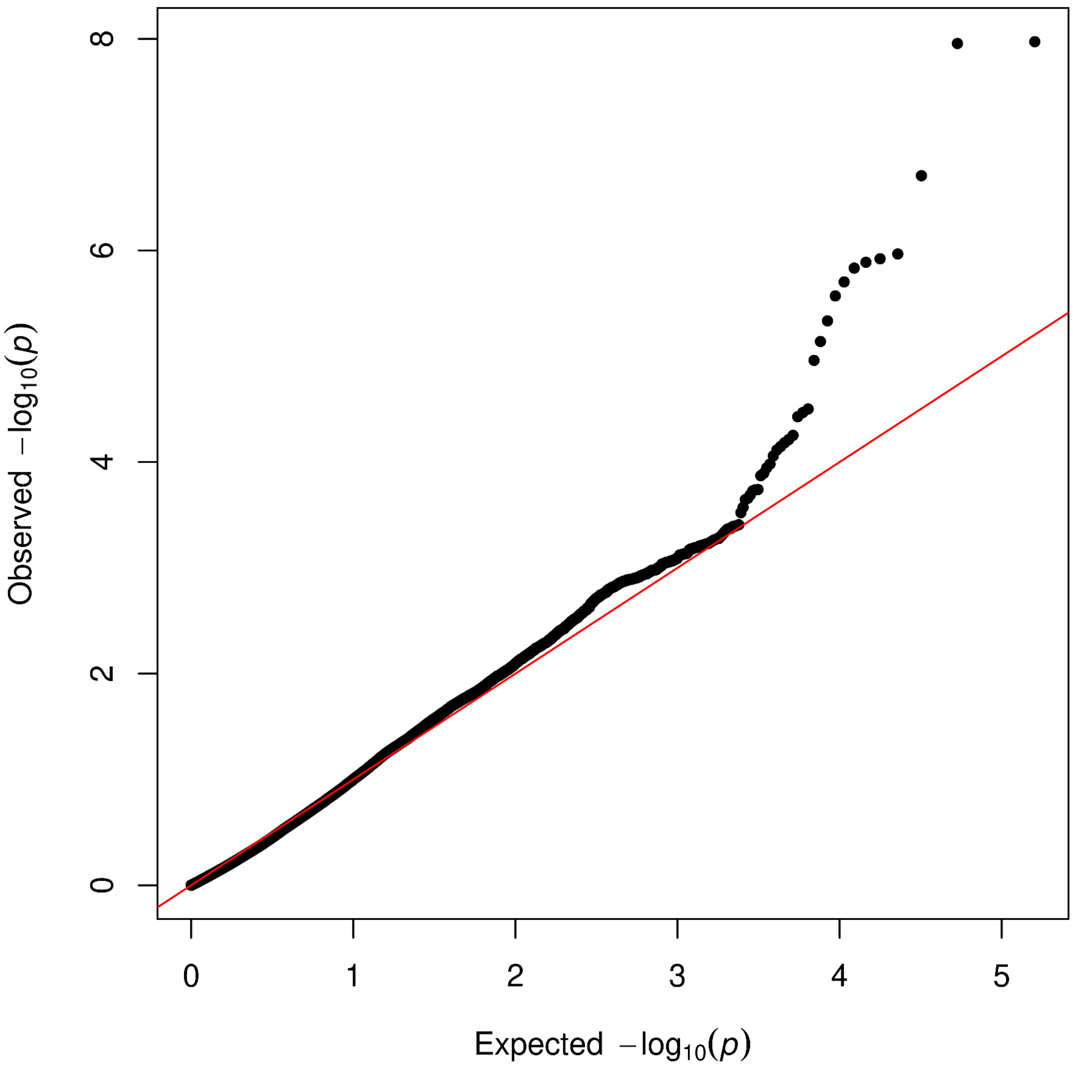

Figure S19. **Quantile-quantile (Q-Q) plot comparing observed and expected $-\log_{10}(p)$ values from the GLMM-based GWAS for body height (large individuals).** *The red diagonal line represents the null expectation under no association; deviations from this line indicate inflation or true association signals.*

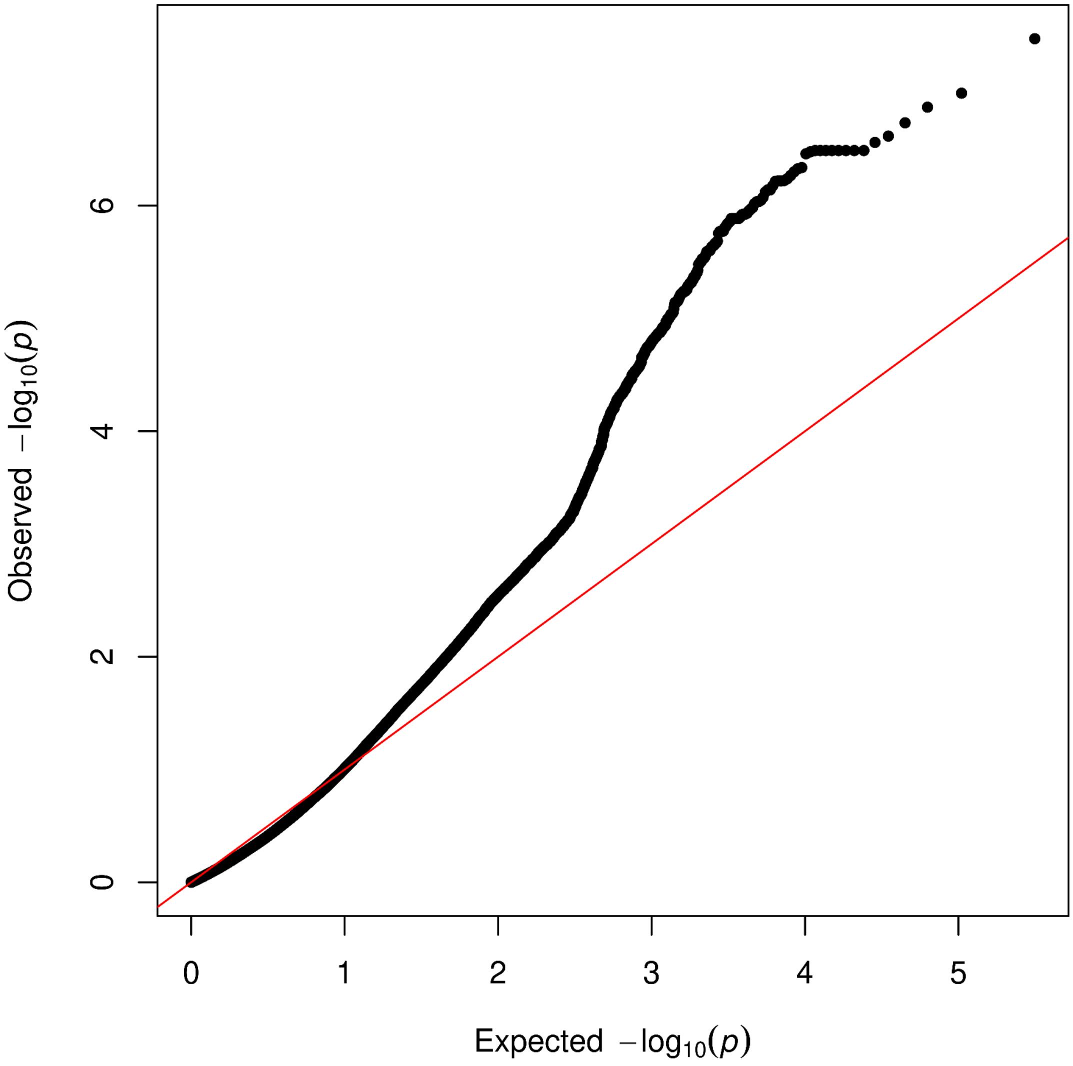

Figure S20. Quantile-quantile (Q-Q) plot comparing observed and expected $-\log_{10}(p)$ values from the GLMM-based GWAS for body weight (all individuals). *The red diagonal line represents the null expectation under no association; deviations from this line indicate inflation or true association signals.*

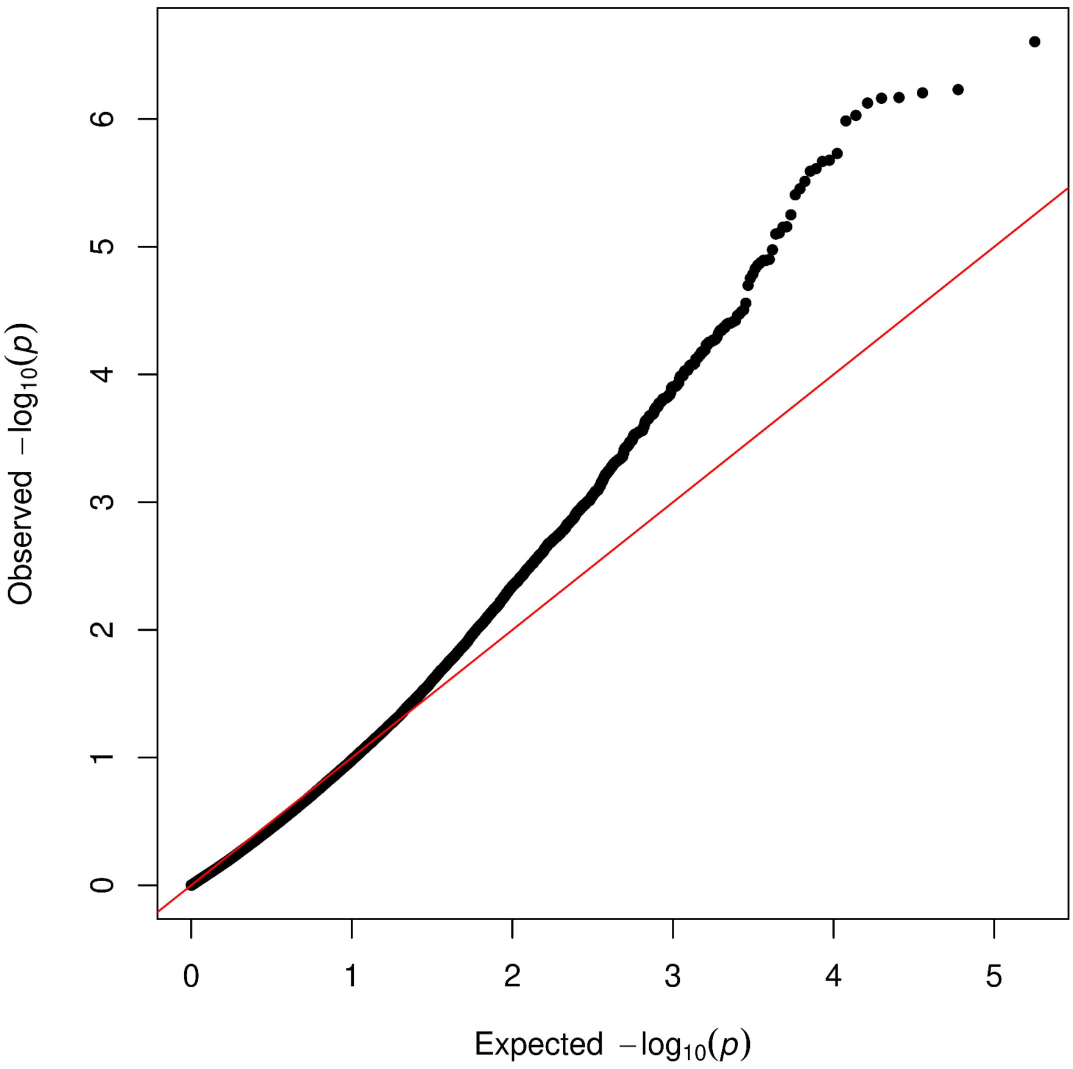

Figure S21. Quantile-quantile (Q-Q) plot comparing observed and expected $-\log_{10}$(p) values from the GLMM-based GWAS for body weight (small individuals). *The red diagonal line represents the null expectation under no association; deviations from this line indicate inflation or true association signals.*

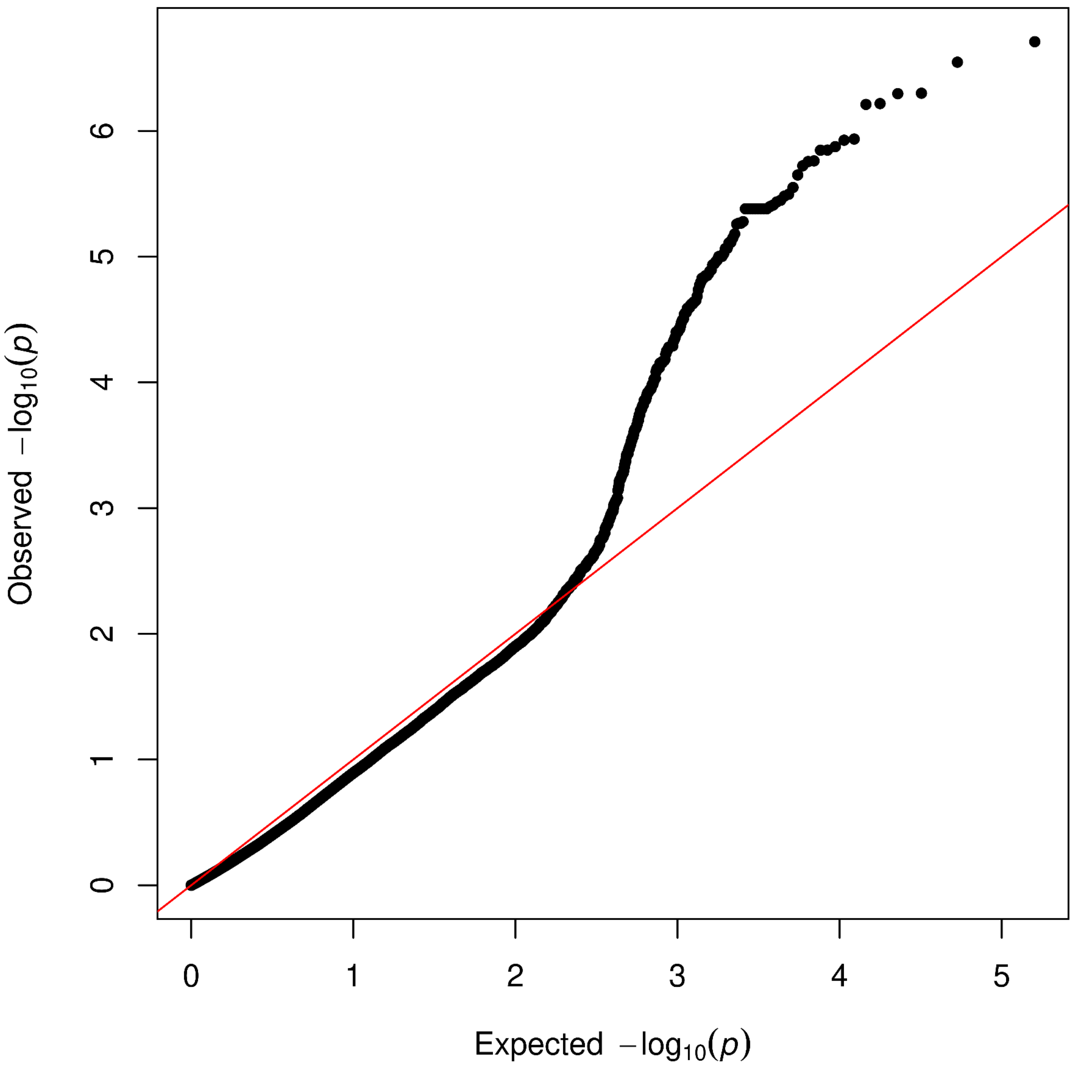

Figure S22. Quantile-quantile (Q-Q) plot comparing observed and expected $-\log_{10}(p)$ values from the GLMM-based GWAS for body weight (large individuals). *The red diagonal line represents the null expectation under no association; deviations from this line indicate inflation or true association signals.*

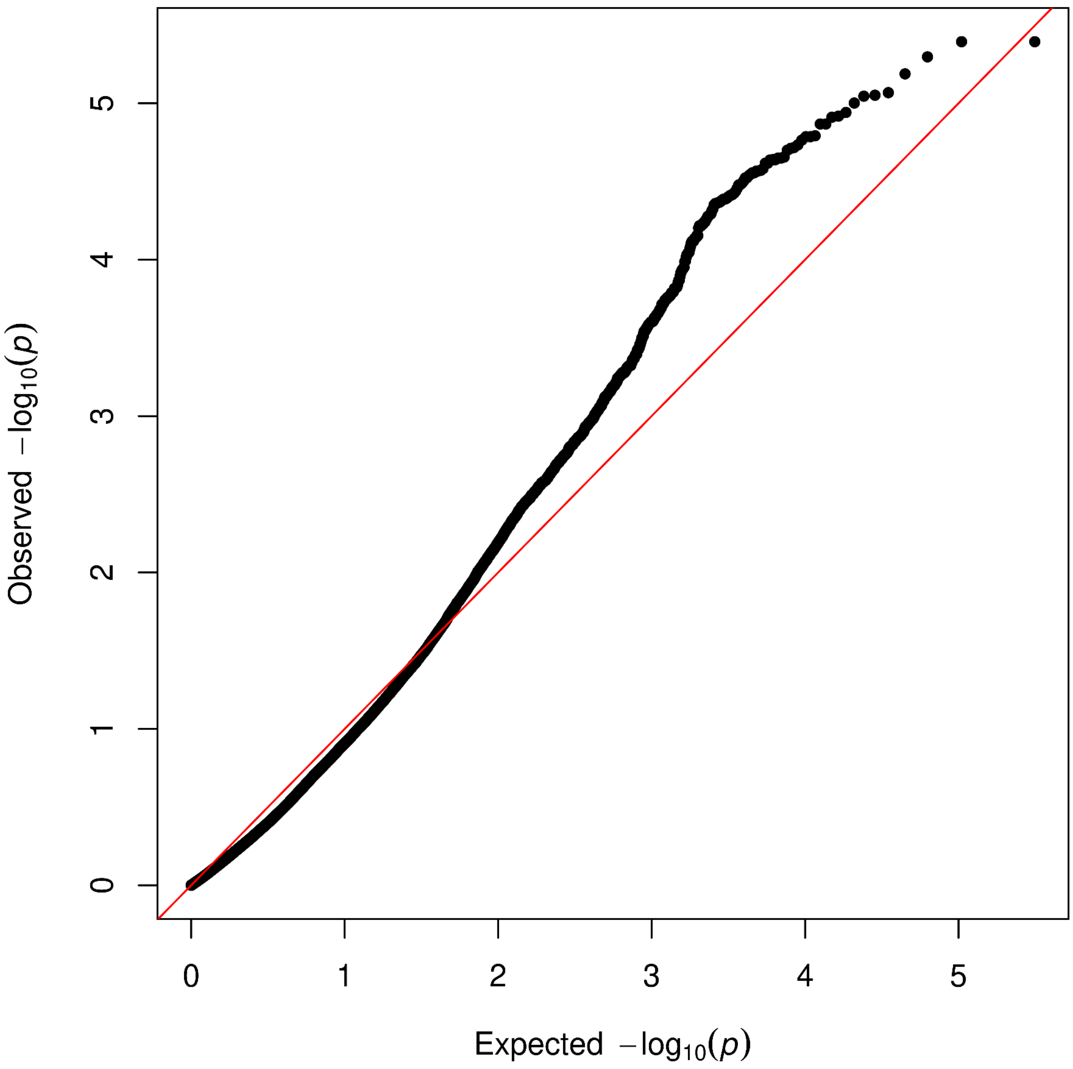

Figure S23. Quantile-quantile (Q-Q) plot comparing observed and expected $-\log_{10}$(p) values from the GLMM-based GWAS for lifespan (all individuals). *The red diagonal line represents the null expectation under no association; deviations from this line indicate inflation or true association signals.*

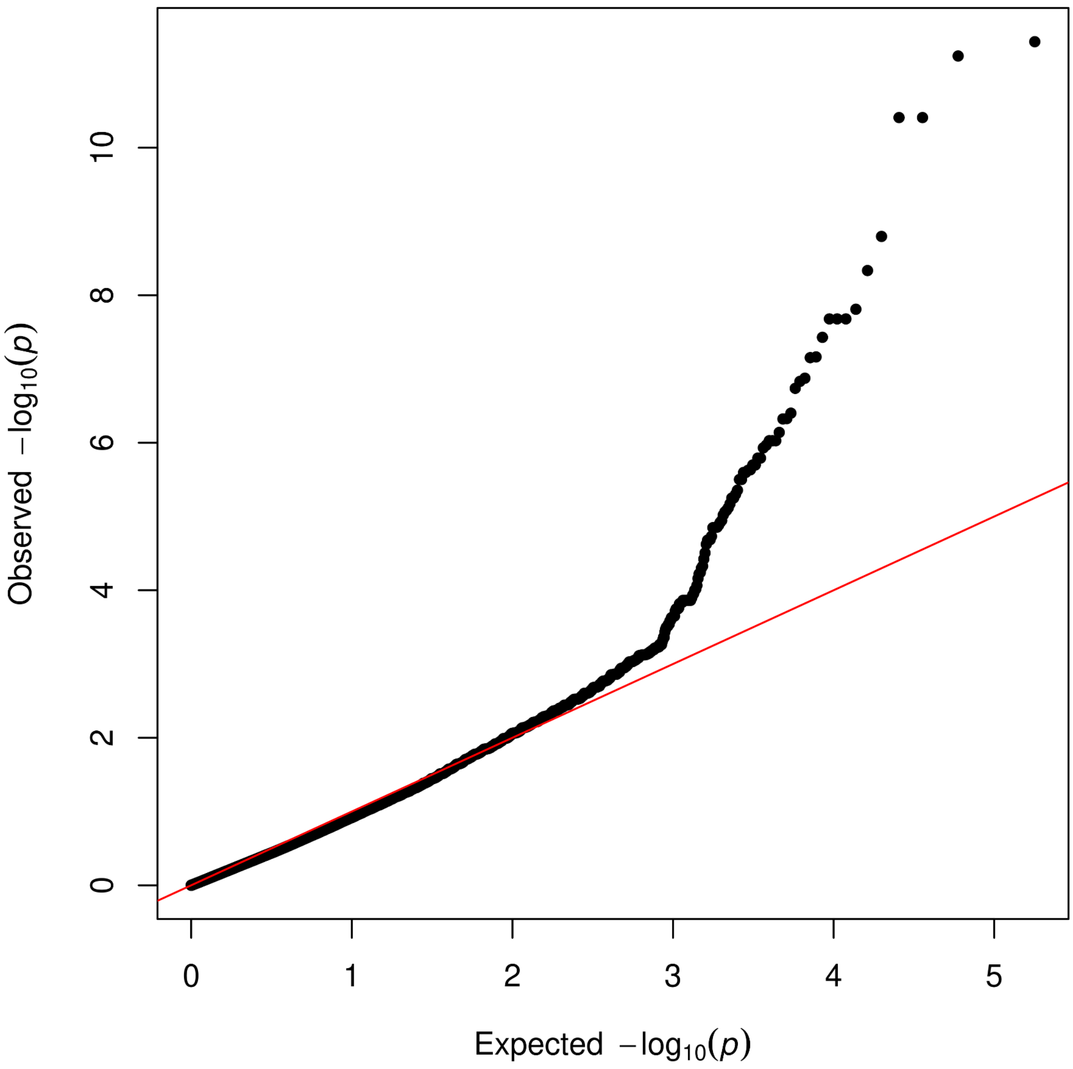

Figure S24. **Quantile-quantile (Q-Q) plot comparing observed and expected $-\log_{10}$(p) values from the GLMM-based GWAS for lifespan (small individuals).** *The red diagonal line represents the null expectation under no association; deviations from this line indicate inflation or true association signals.*

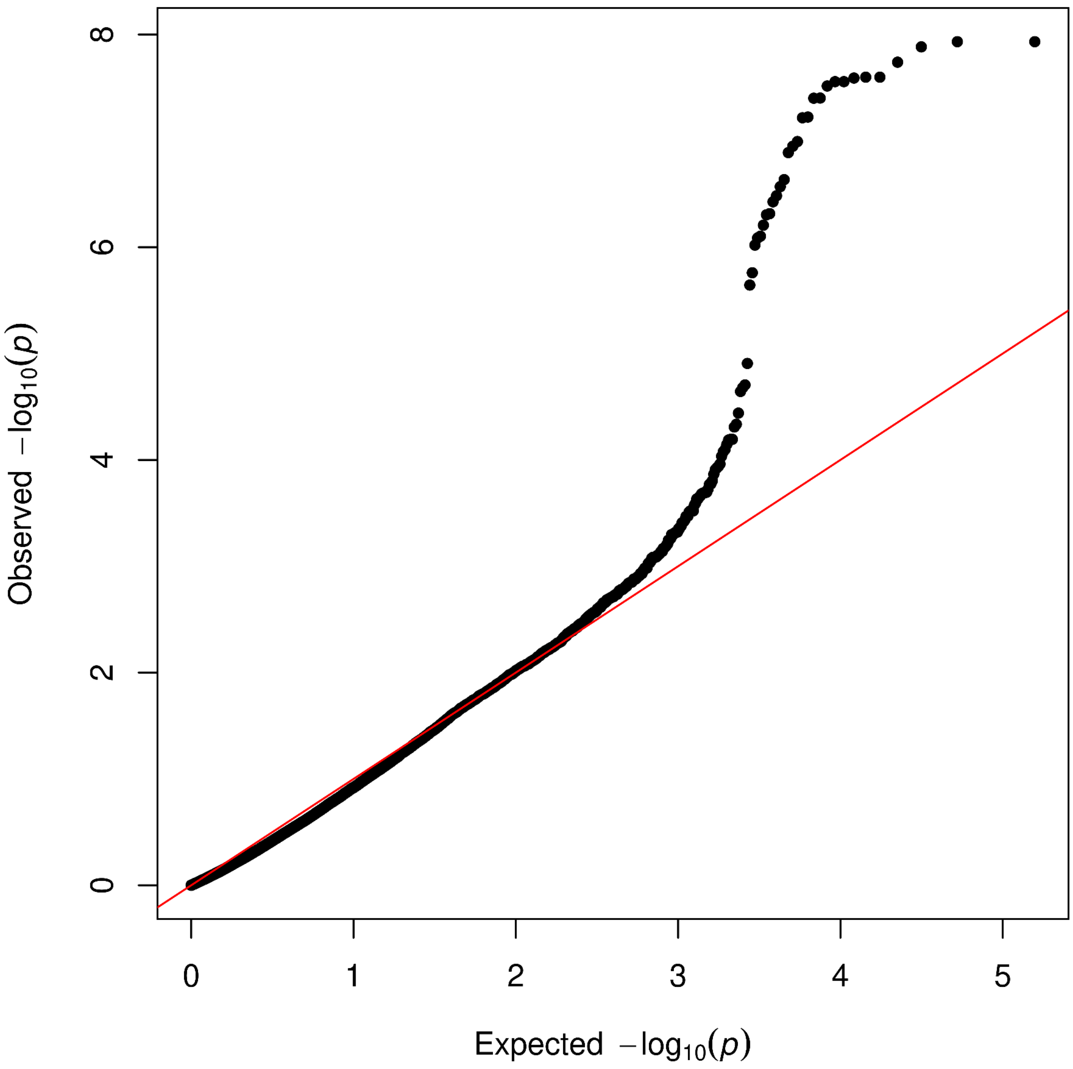

Figure S25. Quantile-quantile (Q-Q) plot comparing observed and expected $-\log_{10}(p)$ values from the GLMM-based GWAS for lifespan (large individuals). *The red diagonal line represents the null expectation under no association; deviations from this line indicate inflation or true association signals.*

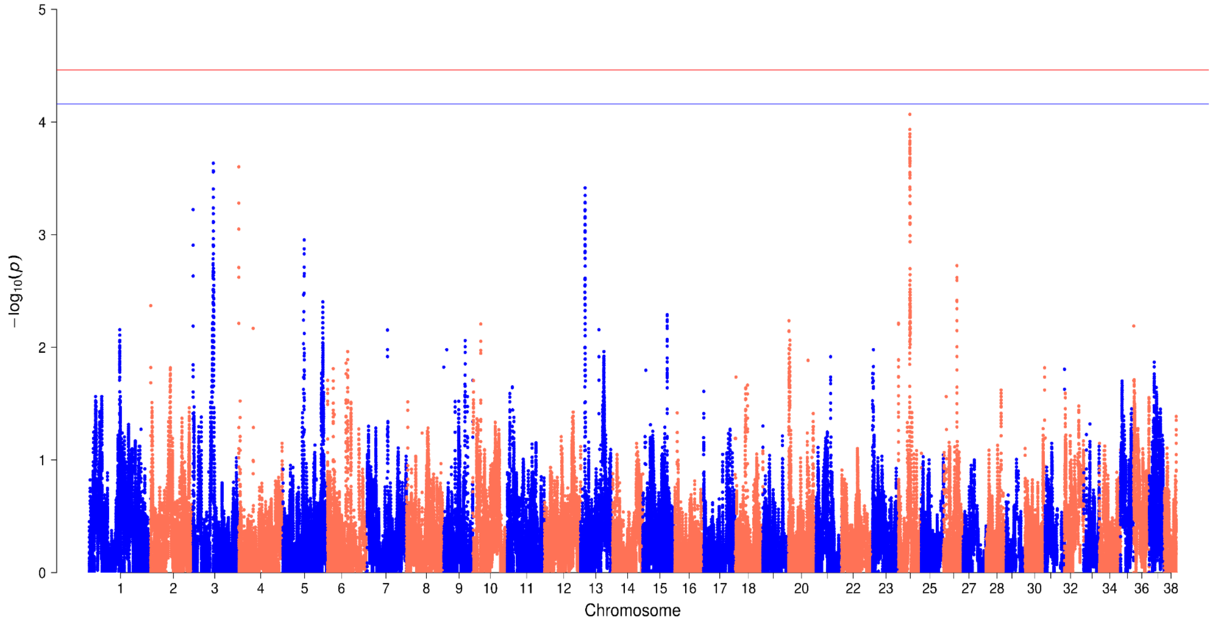

Figure S26. Manhattan plot of GLMM-based GWAS using presence/absence of ROH for the furnish phenotype. *The x-axis shows the genomic position across autosomes, and the y-axis shows the* $-\log_{10}(p)$ *values. Each point represents a single nucleotide polymorphism (SNP). The red horizontal line indicates the genome-wide significance (GWS) threshold, and the blue horizontal line indicates the suggestive-wide significance (SWS) threshold.*

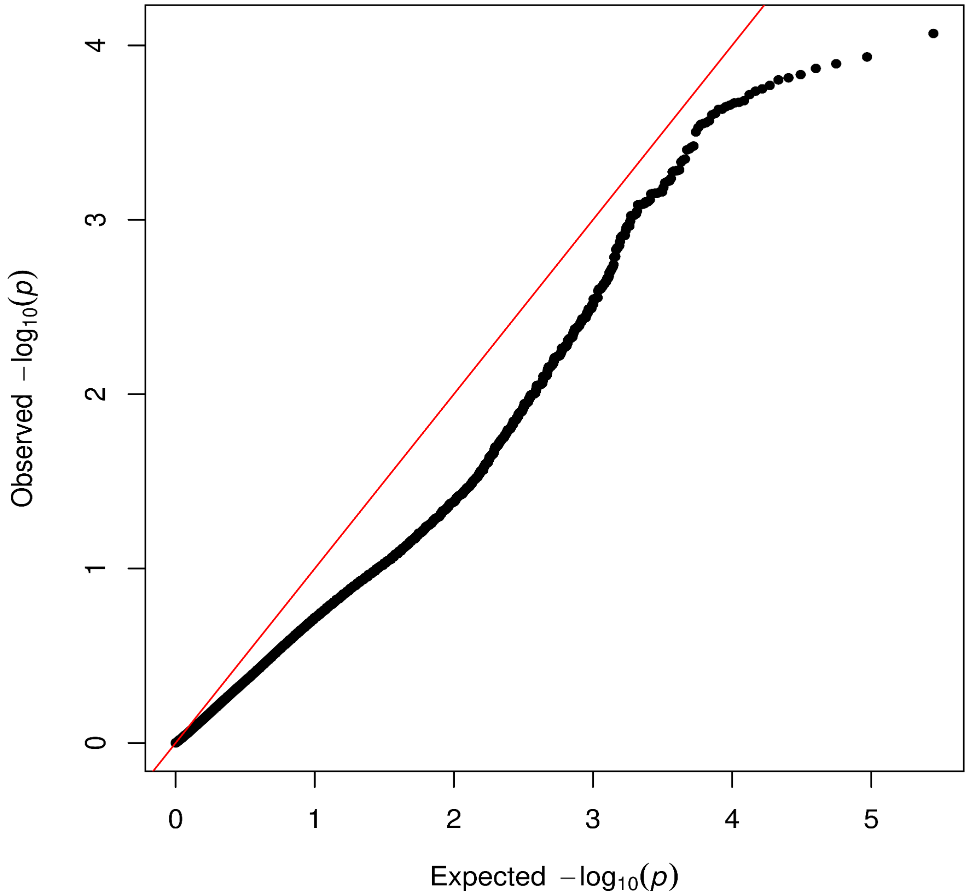

Figure S27. Quantile-quantile (Q-Q) plot comparing observed and expected $-\log_{10}(p)$ values from the GLMM-based GWAS for furnish. *The red diagonal line represents the null expectation under no association; deviations from this line indicate inflation or true association signals.*

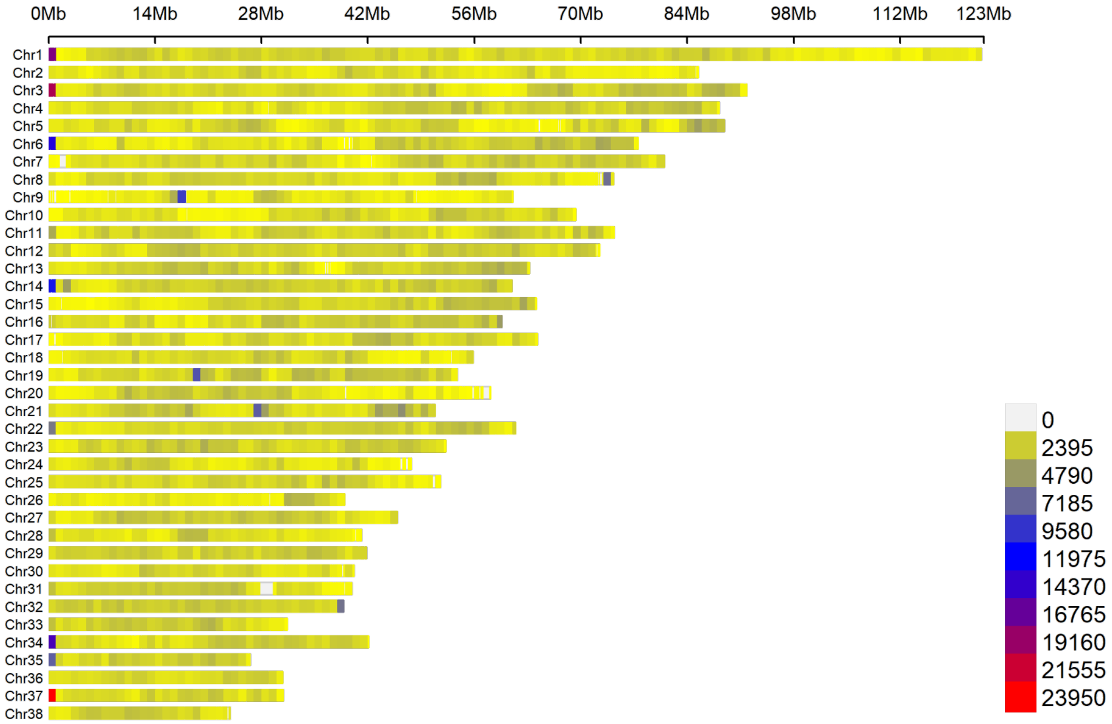

Figure S28. SNP density plot across autosomes (chromosomes 1-38) calculated in 1 Mb windows. *The x-axis shows genomic position (Mb) and the y-axis lists chromosomes. Color scale indicates the number of SNPs per window, with warmer colors representing higher SNP density.*

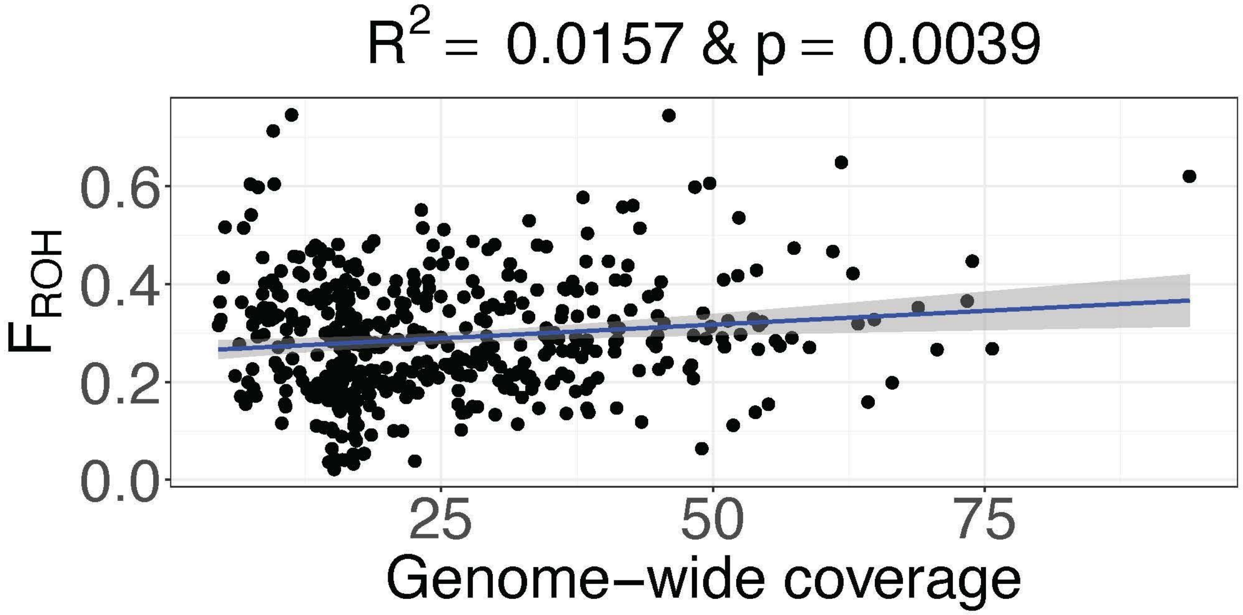

Figure S29. Relationship between genome-wide coverage and F_{ROH} across 466 individuals. *The x-axis shows genome-wide coverage, and the y-axis shows F_{ROH}. The blue line represents the fitted linear regression, with the shaded gray region indicating the 95% confidence interval. The coefficient of determination (R^2) and p-value are shown above the plot.*

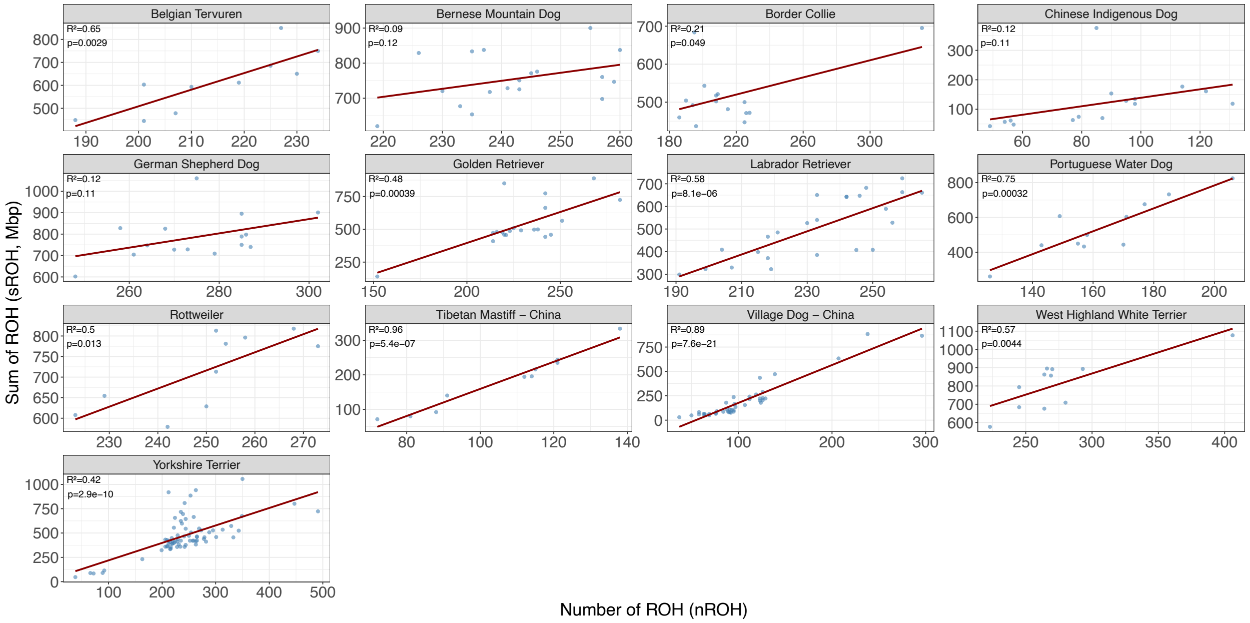

Figure S30. Relationship between n_{ROH} and total ROH length (s_{ROH}, Mb) across breeds with more than 10 individuals. *Each panel corresponds to a breed, with n_{ROH} on the x-axis and s_{ROH} on the y-axis. The red line indicates the fitted linear regression; adjusted R^2 and p-values are shown within each panel.*

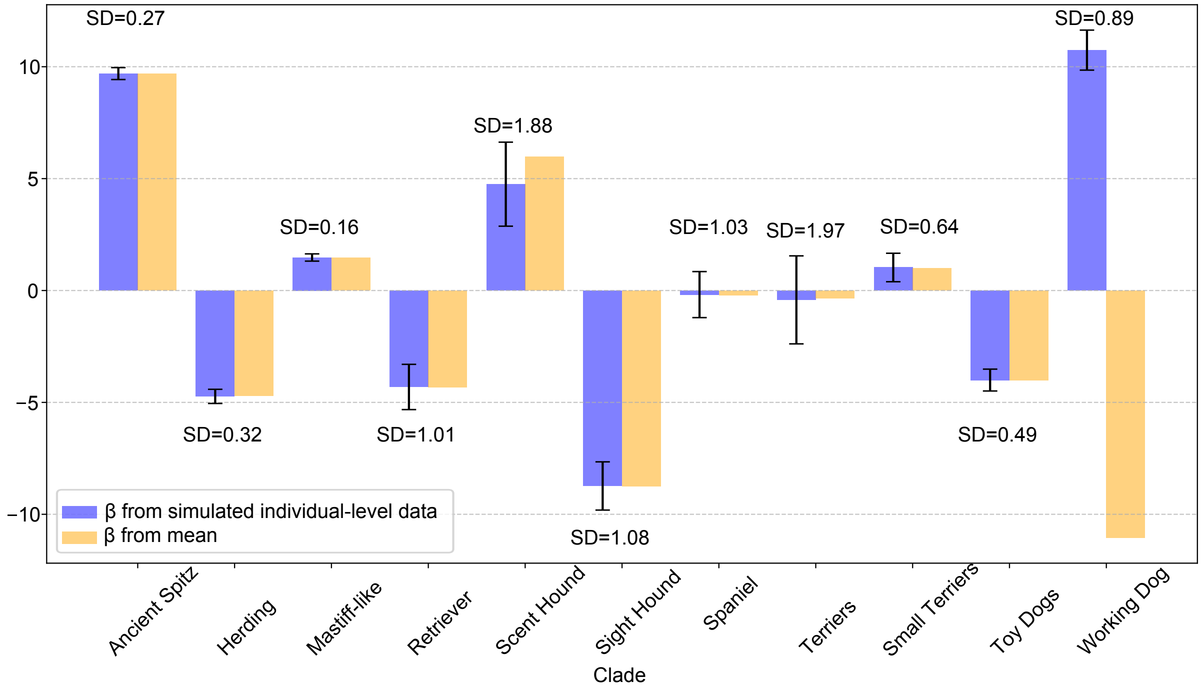

Figure S31. Clade-level effect sizes for lifespan estimated across 1000 simulations. *Blue bars represent beta estimates derived from simulated individual-level data, while orange bars represent beta estimates based on breed-averaged values using empirical* F_{ROH}*. Error bars indicate standard deviation across simulations, with standard deviation (SD) values annotated above each clade.*

Supplementary Text

Assume we have N breeds $b_1, \dots, b_N$ and each breed has M_i dogs, so we have $M_1, \dots, M_N$.
Suppose that the trait score of each breed is normally distributed with $N(\mu_i, \sigma_i^2)$, i.i.d. and F_{ROH} is normally distributed with $N(0, \sigma_i^2)$ across all breeds.
Proof:
Given data (X_{ij_i}, Y_{ij_i}), where:

- i : breed $\in 1, \dots, N$
- j_i : dog index $\in 1, \dots, M_i$ where M_i is # of dogs in breed i.
- $Y_{ij_i} \sim N(\mu_i, \sigma_i^2)$, where σ_i^2 is small for all i, is the trait score of each breed.
- $X_{ij_i} \sim N(0, \sigma^2)$ is the F_{ROH} for all individuals.

We want to show that $Y = \beta_1 X + \beta_0$ (regression from (X_{ij_i}, Y_{ij_i})) is close to $Y = \alpha_1 X + \alpha_0$ (regression from (X_{ij_i}, Y_{ij_i})).
To demonstrate that β_1 is sufficiently close to α_1 we can use MLE regression.
By MLE regression:

- $\beta = \frac{Cov(X_{ij_i}, Y_{ij_i})}{\mathrm{Var}(X_{ij_i})}$
- $\alpha = \frac{Cov(X_{ij_i}, \mu_i)}{\mathrm{Var}(X_{ij_i})}$

Thus, we only need to show $Cov(X_{ij_i}, Y_{ij_i})$ is close to $Cov(X_{ij_i}, \mu_i)$ for all i.
Because $Y_{ij_i} = \mu_i + N(0, \sigma_i^2)$

$$Cov(X_{ij_i}, Y_{ij_i}) = Cov(X_{ij_i}, \mu_i + N(0, \sigma_i^2))$$

By Law of Total Covariance:

$$\begin{aligned} Cov(X_{ij_i}, Y_{ij_i}) &= Cov(E(X_{ij_i} \mid i), E(Y_{ij_i} \mid i)) + E(Cov(X_{ij_i}, Y_{ij_i} \mid i)) \\ &= Cov(X_{ij_i}, \mu_i) + Cov(X_{ij_i}, \mu_i + N(0, \sigma_i^2)) \\ &= Cov(X_{ij_i}, \mu_i) + Cov(X_{ij_i}, N(0, \sigma_i^2)) \\ &\approx Cov(X_{ij_i}, \mu_i) \end{aligned}$$
