# Supplementary material for: Genotypic and phenotypic consequences of domestication in dogs
Source: G3 (Bethesda). 2026 Jul 17;16(9):jkag195. doi: 10.1093/g3journal/jkag195 (PMC13535439; doi:10.1093/g3journal/jkag195)
Supplement: Supplementary file 1 [file jkag195_supplementary_data.zip › Supplemental_Table_Captions_G3-2026-406887.docx]

# **Supplementary Table Captions**

**Table S1. Breed composition and group classification of samples.** *For each breed, the number of individuals included in the analysis is reported along with the corresponding breed group classification.*

**Table S2. ROH calling strategy across breed groups.**  *For each breed group, this table indicates whether runs of homozygosity (ROH) were identified separately within individual breeds or jointly across merged groups of breeds. Breed composition for each category is also shown.*

**Table S3. Quantitative phenotype data.** *Phenotypic values for all quantitative traits (height, weight, and lifespan) are reported for each individual included in the analyses, along with corresponding sample identifiers, breed, and breed group classifications.*

**Table S4. Normalized quantitative phenotype data.** *Normalized values for all quantitative traits (height, weight, and lifespan) are reported for each individual.*

**Table S5. Normalized quantitative phenotype data for small dogs.** *Normalized values for all quantitative traits (height, weight, and lifespan) are reported for each individual in the small dog subset.*

**Table S6. Normalized quantitative phenotype data for large dogs.** *Normalized values for all quantitative traits (height, weight, and lifespan) are reported for each individual in the large dog subset.*

**Table S7. Binary phenotype data for all dogs**. *Binary trait values (0/1) for all phenotypes (bulky, drop ears, furnish, hairless, height, large ears, length of fur, lifespan, long legs, muscled, weight, white chest, and white head) are reported for each individual.*

**Table S8. Association between genomic inbreeding (F_ROH_) and quantitative traits.** *Results from generalized linear mixed models (GLMM) testing associations between F_ROH_ and quantitative traits (height, weight, and lifespan) are shown. Multiple model specifications are included, incorporating F_ROH_ alone, additional covariates (e.g. weight or lifespan), and interaction terms (F_ROH_ x covariate), across different subsets of individuals (all, small, and large). For each model, regression coefficients (β), p-values, and confidence intervals are reported.*

**Table S9. Association between genomic inbreeding (F_ROH_) and binary phenotypic traits.** *Results from generalized linear mixed models (GLMM) testing associations between F_ROH_ and binary traits (bulky, drop ears, furnish, hairless, height, large ears, length of fur, lifespan, long legs, muscled, weight, white chest, and white head) are shown. For each trait, the number of individuals, the log-transformed regression coefficient (*$log\left( \beta\right)$*), confidence intervals, and p-values are reported.*

**Table S10. Genome-wide association analyses of ROH with traits.** *Summary statistics from genome-wide association studies (GWAS) are shown for models testing associations between ROH and traits under different model specifications and sample subsets (all, small, and large). For each model, the genomic inflation factor (λ), genome-wide and suggestive significance thresholds (GWS and SWS), and effective size are reported.*

**Table S11. Summary of runs of homozygosity (ROH) across individuals.** *For each genome, ROH are partitioned into six length classes: Class A (0.5 - 1 Mb), Class B (1 - 2 Mb), Class C (2 - 3 Mb), Class D (3 - 4 Mb), Class E (4 - 5 Mb), and Class F (>5 Mb). For each class, the number of ROH segments (n) and total length (bp) are reported. Total ROH count (nTOTAL) and total ROH length (TOTAL) are also provided, along with summed ROH length (s_ROH_ , Mb) and the genomic inbreeding coefficient (F_ROH_). Breed and breed group classifications are included.*

**Table S12. Summary of ROH counts (n_ROH_) across breed groups and ROH length classes.** *Values represent the mean ± standard deviation of the number of runs of homozygosity within each ROH length class (A-F) for each breed group. The total number of ROH per individual (mean_total_nROH) and the observed range of total ROH counts are also reported.*

**Table S13. Summary of ROH counts (s_ROH_) across breed groups and ROH length classes.** *Values represent the mean ± standard deviation of the summed length of runs of homozygosity (Mb) within each ROH length class (A-F) for each breed group. The total ROH length per individual (mean_total_sROH) and observed range of total ROH lengths are also reported.*

**Table S14. Summary of genomic inbreeding (F_ROH_) across individuals.** *For each sample, the proportion of the genome contained within runs of homozygosity (F_ROH_) is reported alongside the summed ROH length (s_ROH_, Mb), breed, and breed group classification.*

**Table S15. SNPs and genes associated with height in ROH-informed genome-wide association analyses.** *Results from genome-wide association studies (GWAS) of height are shown across model specifications and sample subsets (all, small, and large) within the ROH-based analytical framework. SNPs meeting suggestive or genome-wide significance thresholds are listed, along with their genomic position, p-value* $(-log\left( p \right)$*), and corresponding gene annotations based on transcript coordinates.*

**Table S16. SNPs and genes associated with weight in ROH-informed genome-wide association analyses.** *Results from genome-wide association studies (GWAS) of weight are shown across model specifications and sample subsets (all, small, and large) within the ROH-based analytical framework. SNPs meeting suggestive or genome-wide significance thresholds are listed, along with their genomic position, p-value* $(-log\left( p \right)$*), and corresponding gene annotations based on transcript coordinates.*

**Table S17. SNPs and genes associated with lifespan in ROH-informed genome-wide association analyses.** *Results from genome-wide association studies (GWAS) of lifespan are shown across model specifications and sample subsets (all, small, and large) within the ROH-based analytical framework. SNPs meeting suggestive or genome-wide significance thresholds are listed, along with their genomic position, p-value* $(-log\left( p \right)$*), and corresponding gene annotations based on transcript coordinates.*

**Table S18. SNP density across chromosomes.** *The average number of SNPs per 1Mb window is reported for each chromosome, along with the corresponding standard deviation.*
